# Nano-optical designs for high-efficiency monolithic perovskite–silicon tandem solar cells

**DOI:** 10.1038/s41565-022-01228-8

**Published:** 2022-10-24

**Authors:** Philipp Tockhorn, Johannes Sutter, Alexandros Cruz, Philipp Wagner, Klaus Jäger, Danbi Yoo, Felix Lang, Max Grischek, Bor Li, Jinzhao Li, Oleksandra Shargaieva, Eva Unger, Amran Al-Ashouri, Eike Köhnen, Martin Stolterfoht, Dieter Neher, Rutger Schlatmann, Bernd Rech, Bernd Stannowski, Steve Albrecht, Christiane Becker

**Affiliations:** 1 Division Solar Energy, Helmholtz-Zentrum Berlin für Materialien und Energie GmbH, Berlin, Germany; 2 Computational Nanooptics Group, Zuse Institute Berlin, Berlin, Germany; 3 Soft Matter Physics, Universität Potsdam, Potsdam, Germany; 4 Department of Chemistry, Humboldt Universität zu Berlin, Berlin, Germany; 5 Faculty 1: School of Engineering – Energy and Information, Hochschule für Technik und Wirtschaft Berlin, Berlin, Germany; 6 Faculty of Electrical Engineering and Computer Science, Technische Universität Berlin, Berlin, Germany; 7 Berliner Hochschule für Technik, Berlin, Germany

**Keywords:** Devices for energy harvesting, Optical materials and structures

## Abstract

Perovskite–silicon tandem solar cells offer the possibility of overcoming the power conversion efficiency limit of conventional silicon solar cells. Various textured tandem devices have been presented aiming at improved optical performance, but optimizing film growth on surface-textured wafers remains challenging. Here we present perovskite–silicon tandem solar cells with periodic nanotextures that offer various advantages without compromising the material quality of solution-processed perovskite layers. We show a reduction in reflection losses in comparison to planar tandems, with the new devices being less sensitive to deviations from optimum layer thicknesses. The nanotextures also enable a greatly increased fabrication yield from 50% to 95%. Moreover, the open-circuit voltage is improved by 15 mV due to the enhanced optoelectronic properties of the perovskite top cell. Our optically advanced rear reflector with a dielectric buffer layer results in reduced parasitic absorption at near-infrared wavelengths. As a result, we demonstrate a certified power conversion efficiency of 29.80%.

## Abstract (web summary)

Designing gentle sinusoidal nanotextures enables the realization of high-efficiency perovskite–silicon solar cells

## Main

Monolithic two-terminal perovskite–silicon tandem solar cells (PSTSCs) have recently achieved power conversion efficiencies (PCEs) exceeding 31%^1^, thus overcoming the physical limit of conventional crystalline-silicon single-junction solar cells^2^. Such high PCEs were reached by continuous improvements of the optical and electronic properties of PSTSCs. These improvements include, amongst others, switching the cell polarity for enhanced top-contact transmission^3^ and fine-tuning of various layers to improve the optical performance^4–6^. In addition, various publications have addressed the improvement of the electronic properties by optimizing contact layers^7^, utilizing additives^8,9^ and adjusting the perovskite composition^10^ or deposition^11^. Numerical studies underline the importance of adequate light management by introducing textured device interfaces for high PCEs^12,13^. For silicon solar cells, potassium-hydroxide-etched random pyramidal textures with a size of several micrometres are commonly used for light management. However, they are not compatible without further adaptation with solution-processed perovskite absorbers, which result in the highest reported PCEs of perovskite single-junction solar cells so far^14^. In recent years, different approaches to implement light management textures in PSTSCs have been investigated, either by adapting the perovskite deposition technique but leaving the pyramidal texture unchanged^15–18^, or by adapting the textures such that perovskite solution-processing becomes feasible^9,19–23^ (Supplementary Fig. 1). These studies demonstrated remarkable optical improvements in PSTSCs and provided insights into the morphology and optoelectronic properties of textured perovskite top cells. However, it remains a major challenge to develop an appropriate texture, which is able to balance the persistent trade-off between electronic and optical performance of textured PSTSCs with solution-processed perovskite top cells. In recent studies we already introduced gentle sinusoidal nanotextures with submicrometre feature size as promising candidate for PSTSCs: optical simulations indicated that the PCE can be substantially enhanced compared to planar reference tandem solar cells^24^. We further demonstrated experimentally that such nanotextures provide a feasible light-management approach in both solution-processed perovskite^25^ and silicon^26^ single-junction solar cells, without compromising the optoelectronic quality of the respective absorber.

In this work, we present PSTSCs with a gentle sinusoidal nanotexture connecting the advantages of structuring the silicon surface while preserving the material quality of the solution-processed perovskite. We show that the nanotexturing causes a substantial reduction of reflection losses compared to their planar counterpart, strongly improves the fabrication yield enabled by the excellent film formation properties and increases the open-circuit voltage by 15 mV. To meet the challenge of parasitic absorption losses, we further implement a reflector with a dielectric buffer layer (RDBL) at the rear side of the silicon bottom cell. Combining both approaches, the gentle nanotexture at the front side and the reflector with dielectric buffer layer at the rear side of the silicon bottom cell, we demonstrate a monolithic PSTSC with an independently certified PCE of 29.80%.

## Tandem design

**Fig. 1:**
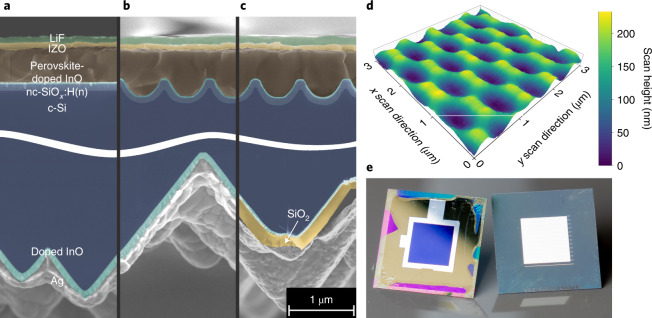
Nanotextured PSTSC design. **a**–**c**, SEM cross-section micrographs of the front and rear side of planar (**a**), nanotextured (**b**) and nanotextured + RDBL (**c**) PSTSCs. c-Si, crystalline silicon. **d**, AFM image of the nanostructured silicon bottom cell front side prior to the deposition of the contact layers. **e**, Photographs of the final PSTSC with a blue active area in between the front-side silver ring of approximately 1 cm^2^ (left) and the RDBL on the rear side (right). Source data

Figure 1a–c shows cross-sectional scanning electron microscopy (SEM) images of the investigated PSTSC device configurations: the reference PSTSC device (Fig. 1a) features a fully planar front side (upper half) and a standard random pyramid texture at the rear side of the silicon heterojunction (SHJ) subcell (lower half). The reference solar cell is similar to that presented in our previous publications^7^ and consists of a SHJ solar cell with n-doped hydrogenated nanocrystalline silicon oxide (nc-SiO_*x*_:H(n)), doped indium oxide as the transparent conducting oxide (TCO), a self-assembled monolayer (SAM) denoted Me-4PACz ([4-(3,6-dimethyl-9H-carbazol-9-yl)butyl]phosphonic acid), a mixed-cation and mixed-halide perovskite absorber, lithium fluoride (LiF), C_60_, tin oxide and indium zinc oxide. The perovskite composition is either Cs_0.05_(FA_0.79_MA_0.21_)_0.95_Pb(I_0.79_Br_0.21_)_3_ or (Cs_0.05_(FA_0.77_MA_0.23_)_0.95_Pb(I_0.77_Br_0.23_)_3_ with bandgap energies of 1.66 eV or 1.68 eV, respectively.

The sinusoidal nanostructure at the front surface of the silicon subcell (Fig. 1b,c) was manufactured by combining ultraviolet nanoimprint lithography, reactive ion etching and wet chemical etching^26^. The experimental procedure is detailed in Methods. The resulting nanotexture has a hexagonal lattice with a period of 750 nm and a peak-to-valley height of approximately 300 nm (Fig. 1d). This nanotexture can be completely covered by the perovskite film with a typical thickness of 500–600 nm (on planar surfaces) revealing a flat perovskite front surface on the C_60_ side. At the rear side of the silicon wafer we applied a reflector with an RDBL^27^ (Fig. 1c). The RDBL comprises a SiO_2_ buffer layer between the TCO and the silver back-reflector, reducing parasitic absorption losses. A silver grid covering 4% of the active area is screen-printed on top of the TCO before SiO_2_ deposition to establish the electric contact between the TCO and Ag (Fig. 1e, right side).

## Perovskite film formation and morphology

**Fig. 2:**
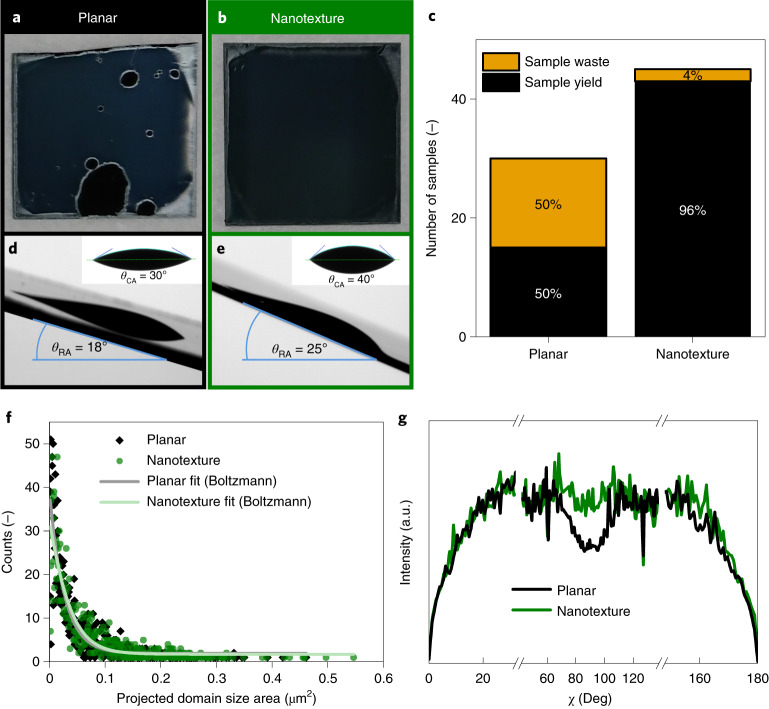
Perovskite film formation and morphology. **a**,**b**, Representative spin-coated perovskite films on planar (**a**) and nanotextured (**b**) silicon bottom cells covered by Me-4PACz (approximate area, 25 × 25 mm^2^). **c**, Fabrication yield of PCTSCs processed on planar and nanotextured silicon bottom cells. **d**,**e**, Side-view photographs of perovskite solution droplets on planar (**d**) and nanotextured (**e**) silicon substrate with roll-off angle (RA) and static contact angle (CA; inset) indicated. **f**, Projected area of domain sizes of perovskite layers deposited on planar and nanotextured substrates as determined from top-view SEM images (Supplementary Fig. 2). **g**, Azimuthal (χ) intensity profiles of the (100) reflection of perovskite averaged over 39 incidence angles from 0.1° to 2° as determined from GIWAXS measurements. Source data

Although Me-4PACz improves the electronic properties (for example, compared to 2PACz and poly(triaryl amine)), the suboptimal formation of perovskite films on planar bottom cells/Me-4PACz often leads to macroscopic holes (Fig. 2a). However, when the perovskite is spin-coated onto nanotextured silicon bottom cells covered with Me-4PACz (Fig. 2b), the occurrence of macroscopic holes is strongly reduced. After the perovskite was deposited, we visually inspected the samples and rejected any that exhibited macroscopic holes. As illustrated in Fig. 2c, out of 45 processed nanotextured tandem solar cells, only two had visible holes after perovskite spin-coating (∼95% yield). In contrast, out of 30 planar devices, which were processed in parallel to the nanotextured cells, 15 showed macroscopic holes (~50% yield). To obtain a more systematic understanding of this observation, we measured the static contact angle and the roll-off angle of the perovskite solution on the respective surfaces (Fig. 2d,e). We find decreased surface energy and reduced wettability on the nanostructured surfaces compared to the planar surface (see static contact angle measurement as insets). However, reduced wettability does not necessarily lead to a reduction in droplet retention: on the nanotextured sample, we observe a larger roll-off angle (25°), which is defined as the tilting angle at which a droplet of perovskite solution starts to roll off the surface^28^, than for the planar reference (18°). This indicates the improved ability of the nanotextured surface to retain the perovskite solution. This observation can be explained from the approach of de Gennes and co-workers, in which the resulting droplet retention force is enhanced by surface roughness pinning the three-phase contact line^29,30^. Such phenomena are a feature of pseudo-superhydrophobic surfaces, which exhibit very large contact angles, but on which the droplet is pinned by a particular combination of material and texture, resulting in an unusually large roll-off angle^31^. We hence regard increased droplet retention on nanotextured surfaces as an important factor for our improved fabrication yield.

To further study the morphological properties of perovskite layers grown on sinusoidal nanotextures, we captured SEM top-view images (Supplementary Fig. 2), and analysed the projected domain size distribution as depicted in Fig. 2f. The domain size distribution is equal with a mean equivalent disk radius of 130 ± 60 nm and 120 ± 60 nm for planar and nanotextured silicon bottom cells, respectively. This is not surprising as it was reported that with the antisolvent method the crystallization is initiated at the top surface, which is not affected by the buried nanotextured interface^32^.

To gain further understanding of the influence of nanotexture on the morphological properties of the perovskite layers, we performed depth-resolved grazing-incidence wide-angle X-ray scattering (GIWAXS) measurements: representative GIWAXS maps of planar and nanotextured samples are shown in Supplementary Fig. 3. Figure 2g displays the azimuthal intensity profiles of the (100) reflection of perovskite layers deposited onto planar and nanotextured silicon bottom cells, averaged over 39 incidence angles (0.1°–2.0°). The perovskite deposited onto the planar substrate shows less intense diffraction intensities at azimuthal scattering angles (*χ*) of 70°–90°, resembling a previous report for a similar perovskite composition^33^. In contrast, a homogeneous intensity distribution is shown in the perovskite deposited on the nanotextured substrate, which indicates a random crystallite orientation of perovskite in the bulk and surface. Previous studies reported different mechanisms, which can lead to a variation of crystal orientation: it can be controlled by the chemical composition of precursors^33,34^, the underlying layer^35^ or by crystal seeds^36^. It was also reported that nanotexturing of perovskite affects the crystal orientation^37^. We further could not detect a notable difference in the distribution of lead iodide in perovskite layers deposited on planar and nanotextured silicon cells (Supplementary Fig. 4), which could affect the optoelectronic properties^38^.

## Optical analysis

**Fig. 3:**
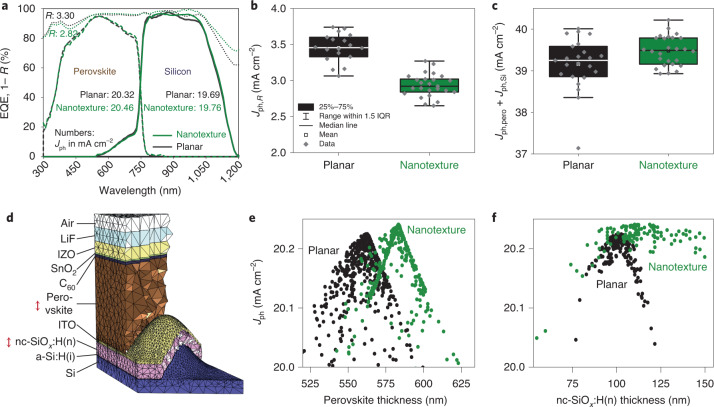
Optical analysis of nanotextures in PSTSCs. **a**, Representative experimental EQE and 1 − *R* spectra for a typical pair of planar (black lines: dotted 1 – *R*; dashed, perovskite EQE; solid, silicon EQE) and nanotextured (green lines: dotted, 1 - *R*; dashed, perovskite EQE; solid, silicon EQE) PSTSCs with standard rear reflector (no RDBL). Numbers are *J*_ph_ in mA cm^−2^. **b**,**c**, Box-plot diagrams of integrated current density from experimental reflection measurements (*J*_ph,*R*_) (**b**) and the sum of integrated current densities in the perovskite and silicon subcell from EQE measurements (*J*_ph,pero_ + *J*_ph,Si_) (**c**). IQR, interquartile range. **d**, Cross section through a meshed unit cell of a PSTSC with nanotextures between perovskite and silicon subcells, as used for FEM simulations. a-Si:H(i), intrinsic hydrogenated amorphous silicon. **e**,**f**, Sensitivity analysis for different layers, based on optical simulations: minimum of photogenerated current densities min[*J*_ph_(pero),*J*_Si_(pero)] as a function of perovskite (**e**) and nc-SiO_*x*_:H(n) (**f**) layer thickness for planar and nanotextured PSTSCs. In **e**, the perovskite thickness for the nanotextured device is defined as the thickness of a planar layer with the same overall volume. For clarity, in **f** only data for samples with a perovskite thickness within ±5 nm of the optimal perovskite thickness are shown. Source data

The optical performance of the planar and nanotextured PSTSCs was analysed by measuring the external quantum efficiency (EQE) and the reflectance (*R*). Representative EQEs and 1 − *R* spectra for planar and nanotextured PSTSCs are shown in Fig. 3a; no notable difference can be observed in the EQEs of the perovskite. In contrast, the EQEs of the silicon subcells differ from each other: the nanotexture diminishes the peaks and valleys caused by thin-film interference (green solid line), which occurs within the perovskite top cell. In addition, the integrated current densities (*J*_ph,Si_) of the silicon EQEs are similar. In contrast to the EQE measurements, nanotexturing reduces reflectance considerably from 3.30 to 2.82 mA cm^−2^ current-density-equivalent. Results from a larger number of processed PSTSC devices confirm an average nanotexture-induced reduction of reflectance of around 0.5 mA cm^−^^2^ current-density-equivalent (*J*_ph,*R*_) (Fig. 3b). Statistical analysis of the combined photogenerated current density (*J*_ph,Pero_ + *J*_ph,Si_) from EQE measurements yields the highest values for selected nanotextured devices, confirming their optical potential. The average values are 39.15 and 39.47 mA cm^−^^2^ for planar and nanotextured devices, respectively, demonstrating that nanotextures enhance optical device performance (Fig. 3c). The partial compensation of optical gain as expected from reflectance might be attributed to collection losses under the low-light conditions of the EQE measurement for selected nanotextured silicon bottom cells (Supplementary Fig. 5). Nonetheless, the overall high level of optical performance was independently confirmed by the EQEs calculated from the relative spectral response measurements at Fraunhofer ISE CalLab (see Supplementary Fig. 6a,b for certificate and EQE spectra) with photogenerated current densities of 20.31 and 19.70 mA cm^−2^ for perovskite and silicon subcells of a nanotextured PSTSC, respectively. Despite the current mismatch, which has little affect on the combined photogenerated current density (Supplementary Fig. 7), the combined photogenerated current density of 40.01 mA cm^−2^ is among the highest values reported in the literature for two-terminal PSTSCs. Higher values have only been demonstrated with fully textured PSTSCs (Supplementary Fig. 8)^15,16,18^.

In view of process robustness we performed a numerical sensitivity analysis to study the influence of layer thicknesses in the top cell on the photogenerated current density. We used data sets obtained during a Bayesian optimization based on the finite-element method (FEM), which rigorously solves Maxwell’s equations. Figure 3d shows a cross section through a meshed unit cell of a nanotextured PSTSC, as used in the simulations. A previous study showed no influence of the period of sinusoidal textures on the reflectivity of PSTSCs for periods ranging from 500 to 1,000 nm (ref. ^13^). This is in contrast to pyramidal textures, which reveal a strong dependence of antireflective effect on pyramid size^20,21^. During the Bayesian optimization, the overall current density of the monolithic tandem device was optimized by maximizing the minimum of the two subcell photocurrent densities. We assume perfect Lambertian light trapping at the rear side of the silicon bottom cell without any parasitic absorption such that the following results mainly concern the perovskite top-cell layer design. The results of the sensitivity analysis are shown in Fig. 3e,f as functions of the perovskite and nc-SiO_*x*_:H(n) layer thickness, respectively. According to these simulations, the optimized nanotextured PSTSC performs almost the same as the optimized planar device with matched photogenerated current densities of 20.24 and 20.22 mA cm^−^^2^, respectively. As seen in Fig. 3e, the sensitivity to a changing perovskite thickness is also similar for the planar and nanotextured designs. However, texturing reduces the sensitivity to a changing nc-SiO_*x*_:H(n) thickness, as seen in Fig. 3f: whereas for planar devices the photocurrent density peaks at around 100 nm nc-SiO_*x*_:H(n) thickness, for the nanotextured design the photocurrent density reaches a plateau for nc-SiO_*x*_:H(n) layers thicker than 100 nm. This means that nanotexturing of the PSTSC widens the process window for the optical nc-SiO_*x*_:H(n) interlayer, which is an important aspect for industrialization of the tandem technology especially when processing on larger areas.

## Optoelectronic analysis

**Fig. 4:**
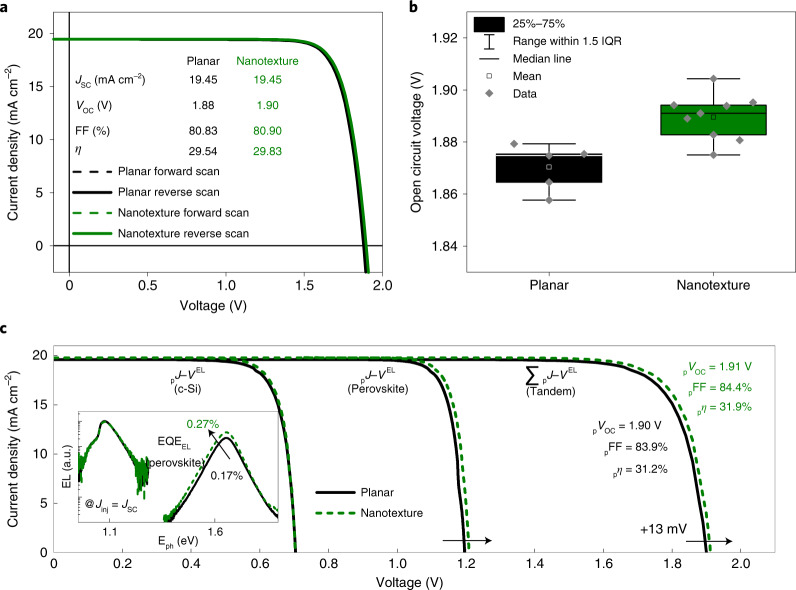
Solar cell characteristics of nanotextured PSTSCs. **a**, Representative current-density/voltage (*J*–*V*) characteristics of planar and nanotextured PSTSCs. **b**, Box plot of the open-circuit voltage for planar and nanotextured PSTSCs with a perovskite bandgap energy of 1.68 eV. **c**, Pseudo-*J*–*V* characteristics of planar and textured PSTSCs showing the individual crystalline silicon and perovskite subcells via subcell-selective EL measurements. Inset: EL spectra at *J*_inj_ = *J*_SC_. Source data

Figure 4a shows the current-density/voltage characteristics of representative nanotextured and planar PSTSCs. Both devices show PCEs (η) above 29% with a *J*_SC_ of 19.45 mA cm^−2^. In addition, the fill factor (FF) of the planar and nanotextured PSTSC shows similar values. Even when considering many devices and the influence of current density mismatch (*J*_ph,Pero_ − *J*_ph,Si_) on the FF (ref. ^6^), no clear difference can be observed between the planar and nanotextured configurations (Supplementary Fig. 9). Alongside the electronic characteristics, we tested the stability at maximum power point by subjecting both a planar and a nanotextured PSTSC to continuous illumination in a dedicated tandem ageing set-up. The observed degradation over time (Supplementary Fig. 10) resembles previous results^7^. We do not observe an effect of nanotextures on the stability of the tandem devices. In contrast, the open-circuit voltages (*V*_OC_) of planar and nanotextured PSTSC differ substantially. Figure 4b displays the *V*_OC_ of planar (black box) and nanotextured (green box) PSTSCs with a perovskite bandgap of 1.68 eV. The overall *V*_OC_ distribution shows higher maximum values and a statistical improvement of the median *V*_OC_ by around 15 mV for nanotextured compared with planar PSTSCs. To verify and understand this effect, we used a subcell-selective characterization approach based on electro- and photoluminescence (EL/PL), which makes it possible to assess the charge transport and recombination properties of the top and bottom cell and to determine their efficiency potential. First, we quantified the injection-dependent electroluminescence quantum yield (EQE_EL_) of both subcells by injecting a current into the tandem device and detecting the emitted EL of both subcells. From the emitted EL, we calculated the quasi-Fermi level splitting (QFLS_EL_). The injection-dependent QFLS_EL_ equals a series-resistance-free dark *J*–*V* curve, from which we can generate a pseudo-*J*–*V* curve by adding the generation current^39^. Figure 4c shows the obtained characteristics of both subcells in the tandem configuration for planar and nanotextured PSTSCs. These results reveal an approximately ~60% enhanced EQE_EL_ in the perovskite subcell for the nanostructured PSTSC (Supplementary Fig. 11), which explains the ~15 mV *V*_OC_ gain in the corresponding *J*–*V* measurements. We note that the pseudo-FF (_p_FF) of 84.5% and 84.1% for nanotextured and planar PSTSCs, respectively, clearly surpass the experimental values and thus point to a further optimization route for these solar cells (see also the results of intensity-dependent *J*–*V* measurements in Supplementary Fig. 12). Further, the EL emission of perovskite in the nanotextured devices is slightly skewed (Fig. 4c, inset), which might suggest enhanced photon recycling^40^. We note that the implied performance of both silicon subcells is identical and that the efficiency potential obtained with the nanostructured device is almost 32%. The exact mechanism that improves the EQE_EL_/*V*_OC_ of the perovskite on the nanotextured silicon cell is not yet fully understood. The enhancement might stem from an improved optoelectronic quality of the absorber layer, possibly related to alterations in the crystal orientation (Fig. 2g). It is known that the impact of crystallite orientation on the carrier dynamics of layered perovskite is crucial; however, this relation has been the subject of controversy for three-dimensional perovskite^41^. There are both research reports claiming that the improved crystallite orientation contributes to improved optoelectronic properties^33,42,43^, and those claiming that there is no clear correlation^44^. In addition, slightly lower non-radiative recombination losses at the interfaces^45^, or an alteration of the optical coupling between the perovskite and the silicon^46^ have also been discussed. In a study by Hou et al., a *V*_OC_ enhancement of the perovskite on textured silicon was related to an alteration of the electric fields in the perovskite with extended drift regions^21^. We observe no remarkable difference in the dark currents of nanotextured and planar solar cells, and almost no difference in the morphology (Fig. 2), hence the higher emission from EL (Fig. 4) indicates less non-radiative recombination in nanotextured devices.

## High-performance tandem solar cells with optically advanced rear reflector

**Fig. 5:**
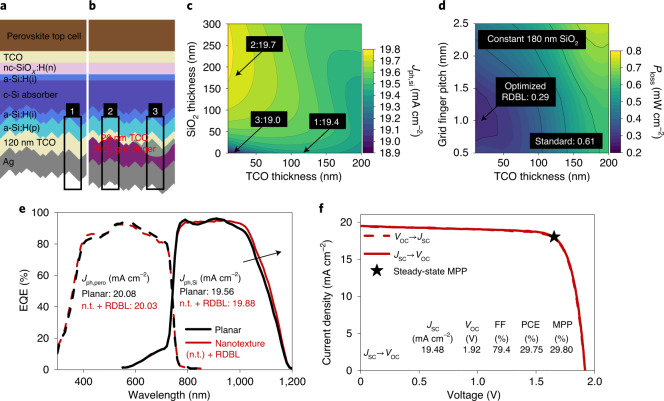
Implementing a rear RDBL for high-perfomance nanotextured PSTSCs. **a**,**b**, Schematic of the PSTSC layer stack with standard reflector (**a**) and with an RDBL at the rear side (**b**). (i), (n) and (p) refer to intrinsic, n-doped and p-doped layers. The numbered black frames mark the standard rear side (1) and the SiO_2_ (2) and grid finger (3) regions of the RDBL. **c**, 2D map of *J*_ph,Si_ for different SiO_2_ (*y* axis) and TCO thicknesses (*x* axis). The configurations corresponding to the experimental layer stacks are marked with arrows and the corresponding *J*_ph,Si_ is indicated. **d**, 2D map of power losses in the silicon bottom cell for different grid finger pitches (*y* axis) with 40 µm finger width and TCO thickness (*x* axis). Power loss in mW cm^−^^2^ is shown for the RDBL configuration and for the standard reflector. **e**, EQE spectra (dashed lines, perovskite EQE; solid lines, silicon EQE) of a planar PSTSC with a standard rear side (black) and a nanotextured (n.t.) PSTSC with an RDBL (red). The integrated photogenerated current densities of perovskite (*J*_ph,pero_) and silicon (*J*_ph,Si_) are shown. **f**, Current-density/voltage (*J*–*V*) characteristics of a nanotextured PSTSC with an RDBL certified by CalLab at Fraunhofer ISE. Source data

To reduce parasitic absorption losses in the rear reflector, we additionally implemented an RDBL^27,47,48^. State-of-the-art monofacial silicon heterojunction solar cells use a TCO/silver system as reflector. By designing a thin TCO layer and at the same time increasing the distance between metal and silicon by means of an RDBL, parasitic absorption losses can be reduced. Bush et al. integrated such a reflector into a PSTSC, thereby increasing the *J*_SC_ and efficiency considerably^49^. Figure 5a,b illustrates PSTSC layer stacks with a standard reflector and with an RDBL. Three optically different regions can be distinguished: (1) standard reflector (120 nm TCO, no buffer layer), (2) RDBL (20 nm TCO, 180 nm SiO_2_ buffer layer) and (3) grid fingers of the RDBL (20 nm TCO, no buffer layer). SEM images of the corresponding regions are presented in Supplementary Fig. 13. The SiO_2_ layer thickness and the grid finger pitch were chosen according to optical and electrical simulations of the RDBL. To quantify the optical properties of these three regions, we performed simulations of the variation of the photogenerated current density in the silicon (*J*_ph,Si_) with TCO and SiO_2_ thickness (Fig. 5c). For the optimized layer thicknesses, the photocurrent density of the silicon bottom cell *J*_ph,Si_ increases from 19.4 to 19.7 mA cm^−2^ when replacing the standard reflector design (region 1) with the RDBL (region 2), mostly due to reduced parasitic absorption in the TCO–silver contact. A full-area design as in the grid finger (region 3) would lead to a *J*_ph,Si_ of 19.0 mA cm^−2^.

Finally, the local contact area has to be optoelectronically optimized not only regarding the optical properties, but also in terms of lateral transport and contact resistive losses. This means that the TCO thickness and the grid finger geometry have to be balanced. Figure 5d shows the simulation results (see Methods for details). We find decreased power loss with TCO thickness reduction, since the benefit from reduced parasitic absorption dominates. For a silver-grid finger width of 40 µm we find an optoelectrical optimum for a grid pitch of roughly 1.1 mm (Fig. 5d). Hence, a finger pitch of 1 mm was implemented, resulting in local contacts covering roughly 4% of the active area. The optoelectronic analysis further shows that overall the RDBL potentially increases the power output by 0.3 mW cm^−2^. The EQE spectra of experimentally realized PSTSCs with and without an RDBL are shown in Fig. 5e. In addition to the nanotexture-induced flattening of the silicon EQE, the RDBL features an increased absorption at the silicon band edge, as expected from the optical simulations. To account for the additional current density from the RDBL in the silicon subcell, we shifted the perovskite bandgap from 1.68 to 1.66 eV. As described by Peña-Camargo et al., the optoelectronic properties do not notably change when slightly adjusting the perovskite composition^50^; only the *V*_OC_ changes according to the bandgap. One of the best PSTSCs, featuring both a nanotextured interface between the perovskite and silicon subcell and an RDBL was sent to Fraunhofer ISE CalLab for independent certification (Fig. 5f; see Supplementary Fig. 14 for certificate). The cell reached a PCE of 29.75% when measured from *J*_SC_ to *V*_OC_ (designated area, 1.0163 cm^2^), with a *V*_OC_ of 1.92 V, an FF of 79.4% and a *J*_SC_ of 19.56 mA cm^−2^. The certified PCE, as determined from MPP tracking, is 29.80%.

## Conclusions

In this study, we integrated gentle submicrometre-periodic nanotextures and an improved back-reflector design into monolithic PSTSCs. The nanotextures enable greatly enhanced process yield of the solution-processed perovskite top cell from around 50% in case of planar to about 95% for nanotextured silicon bottom cells. We further observed a reduction of reflection losses amounting to around 0.5 mA cm^−2^ current-density-equivalent. As a result, combined short-circuit current densities in the perovskite and silicon subcells of up to 40.0 mA cm^−2^ are observed, which is one of the highest values reported in the literature for two-terminal PSTSCs. A sensitivity analysis further indicates that the nanotextures substantially improve the performance robustness upon deviations from the optimal nanocrystalline silicon oxide layer thickness—an important aspect with regards to the industrialization of tandem technology and processing on larger areas. The main driver for improved PSTSC performance was an increase of the open-circuit voltage by around 15 mV that stems, according to our subcell-selective EL characterizations, solely from an improved perovskite-top-cell performance. In addition to the nanotextures at the silicon bottom-cell front side, we further implemented an RDBL at the rear side. This design further improves the current density in the silicon bottom cell by around 0.3 mA cm^−2^ by reducing parasitic absorption losses. By combining nanotextures and RDBL in one PSTSC, we achieved a certified PCE of 29.80%. These results pave the way for the wide use of nano-optical designs in high-efficiency perovskite solar cells and other metal halide perovskite optoelectronic devices in the near future.

## Methods

### Device fabrication

#### Nanotextured silicon bottom cells

A three-step process, developed by Sutter et al^26^., was used to create nanotextures in silicon wafers for PSTSCs. Sinusoidal master structures, manufactured by laser interference lithography^51^, were replicated by nanoimprint lithography with a polydimethylsiloxane stamp in an ultraviolet-curable resist (mrNIL210–500; Microresist Technologies) on a double side polished (100) silicon n-type float-zone wafer (Topsil) with a nominal resistivity of 1–5 Ω·cm and an approximate thickness of 280 μm. For the nanoimprint lithography process, the 4 inch wafers were cleaned according to the standard RCA procedure^52^. In ambient conditions, 700 μl of resist was spin-coated for 30 s at 3,200 r.p.m. on the wafer, prebaked for 3 min at 60 °C and cured by ultraviolet light with the polydimethylsiloxane stamp comprising the inverted nanotexture from the master on top for 2 min. In a second step, the reproduced nanotexture was anisotropically etched into the silicon substrate by reactive-ion etching with the etching gases sulfur hexafluoride and fluoroform (CHF_3_) for 16 min at 90 W radiofrequency power and 20 mtorr pressure. In this step, the nanotextured resist served as a 3D etching mask and was able to replicate the texture into the underlying silicon. An additional reactive-ion etching oxygen plasma was applied to eliminate organic residues. Due to the diffusion of ions and silicon lattice distortion, a subsequent wet-chemical defect etch with nitric acid, phosphoric acid and fluoric acid (HNO_3_(65%)/H_3_PO_4_(85%)/HF(50%)/H_2_O, 30:10:1:15) was performed. Prior to the texturing of the rear side with random pyramids with (111) facets (potassium hydroxide etched, 8 min at 83 °C, CellTex Ultra (ICB) as additive), the nanotextured front side was protected with a 320-nm-thick SiO_*2*_ layer deposited by plasma-enhanced chemical vapour deposition (PECVD). After removal of the capping in HF, a final RCA clean and HF dip (1% dilution in water) were performed. Intrinsic and doped hydrogenated amorphous (a-Si:H(i)/a-Si:H(p)) and nc-SiO_*x*_:H(n) layers were deposited by PECVD using an Applied Materials AKT1600 cluster tool. To account for the enhanced surface area, the process parameters for the front side of the nanotextured wafers were adjusted by a factor of 1.3 versus the planar case. A 20 nm layer of doped indium oxide as TCO was sputtered as the recombination contact. The rear-side contact of the silicon bottom cells is 120 nm for the standard design and 20 nm sputter-doped indium oxide for the RDBL. The RDBL has an additional silver grid printed on the rear side with a nominal finger width of 40 µm and a 1 mm pitch, and was cured for 10 min at 210 °C, followed by a PECVD deposition of 180 nm SiO_2_. For both standard and RDBL rear-side designs, 400 nm silver was sputtered as the back contact layer. In the standard design the silver forms a direct contact with the TCO. For the RDBL design the local contacts are fabricated at the finger grid printed area through the 180 nm SiO_2_ to the silver layer without additional processing steps being needed. All contacts of the silicon bottom cell were processed through a quadratic mask with an area of 1.1 cm^2^. After sputtering silver, the silicon wafers of the standard design were annealed for 10 min at 210 °C and wafers of both designs were additionally annealed for 5 min at 210 °C under 1 sun illumination. The wafers were then laser cut into 2.5 × 2.5 cm^2^ pieces, with a 1.1 cm^2^ contact area in the centre.

For fabricating the perovskite top cells, we adapted a device stack reported earlier^7^. For cleaning, the 2.5 × 2.5 cm^2^ silicon bottom cells were blown with nitrogen, and ethanol was spin-coated at 2,500 r.p.m. for 30 s, folllowed by ultraviolet/ozone treatment for 15 min. Further processes were carried out in a nitrogen-filled glovebox. First, 100 µl of a 3 mM solution of Me-4PACz (TCI) was dissolved in ethanol and spin-coated (3,000 r.p.m. for 30 s) onto the bottom cells, followed by a 10 min annealing at 100 °C. Then, either Cs_0.05_(FA_0.79_MA_0.21_)_0.95_Pb(I_0.79_Br_0.21_)_3_ (1.66 eV bandgap) or Cs_0.05_(FA_0.77_MA_0.23_)_0.95_Pb(I_0.77_Br_0.23_)_3_ (1.68 eV bandgap) perovskite was prepared, adapted from a recipe by Saliba et al^53^. For this, precursor solutions containing formamidinium iodide (Dyenamo) and lead iodide (TCI) were mixed in a ratio of 79:21 or 77:23 with a precursor solution containing methylammonium bromide (Dyenamo) and lead bromide (TCI). Both precursor solutions contained 1 M of the corresponding organic and 1.1 M of the lead salts which were dissolved in a mixture of anhydrous dimethylformamide and dimethylsulfoxide (4:1 vol/vol; both purchased from Sigma Aldrich). Next, 5 vol% caesium iodide (abcr) from a 1.5 M stock solution in DMSO was added to the precursor solutions. The resulting perovskite solution was then spin-coated at 3,500 r.p.m. for 40 s; 15 s prior to the end of the programme, 500 µl ethyl acetate (Sigma Aldrich) was poured onto the spinning substrate. The films were annealed at 100 °C for 30 min. The front-side contact was deposited through subsequent thermal evaporation of 1 nm lithium fluoride (Sigma Aldrich) and 18 nm C_60_ (Sigma Aldrich). Then, 20 nm tin oxide was deposited by thermal atomic layer deposition in an Arradiance GEMStar reactor at 80 °C. A 100 nm layer of indium zinc oxide from a 4 inch target (90:10 wt% In_3_O_2_:ZnO) was deposited by sputtering at a power of 150 W under argon/oxygen. A 100 nm thick silver frame was thermally evaporated through a shadow mask. Finally, 100 nm LiF was thermally evaporated as an antireflective coating. The active area is defined by the metal frame and is slightly larger than 1 cm^2^.

### Characterization

Roll-off measurements were conducted using a contact angle goniometer (DSA 25, Krüss). In all measurements, 10 µl droplets of perovskite solution (see above) were used. For the roll-off angle measurements the samples were placed on fixed, inclined stages before dispensing the solution. The methodology used here enables qualitative analysis of the droplet retention behaviour of both planar and textured surfaces^54^.

Reflectance was measured under an angle of 8° in 5 nm steps from 300 to 1,200 nm with a PerkinElmer Lambda 1050+ ultraviolet/visible/near-infrared spectrophotometer, which was calibrated with a Spectralon. For statistics on the reflectance of PSTSCs, perovskite absorbers with bandgaps varying from 1.66 to 1.68 eV were used.

Atomic force microscopy (AFM) measurements were performed with a XE-70 (Park Systems). For accurate profile scans in the nanometre regime, high-aspect-ratio tips were used. SEM was performed with a Merlin field emission scanning electron microscope with a Gemini II optical column (Zeiss).

Ex situ GIWAXS measurements were conducted at the mySpot beamline at the electron storage ring BESSY II. The samples were measured at room temperature in reflection mode with incidence angles from 0.1° to 2° (39 scans, 0.05° interval), using a radiation energy of 9 keV (*λ* = 1.378 Å). The size of the beam was around 100 µm (ref. ^55^). The 2D images were plotted and integrated with Dpdak and GIXSGUI software^56,57^.

#### EL measurements

Absolute EL measurements were performed using a calibrated silicon photodetector and a Keithley 485 picoammeter. The detector (active area, ∼1 cm^2^) was placed directly in front of the device, and the total photon flux was determined from the emission spectra of the perovskite and silicon subcell, and the EQE of the detector. To selectively pick up EL from the perovskite or crystalline silicon subcell, appropriate long-pass and short-pass filters were used. Underestimation of the EQE_EL_ due to undetected photons that escaped to the side was compensated by additional measurements at different distances and with a larger detector (active area, ∼2 cm^2^). During a typical EL measurement, a Keithley 2400 source meter was used to apply a forward bias to the cell, and the injected current was monitored. Measurements were conducted with a home-written LabVIEW routine. Relative EL spectra of the PSTSCs were measured with an Andor SR393i-B spectrometer equipped with an Andor iDus silicon charge-coupled device camera and an iDus InGaAs detector array. The spectral response of the system was calibrated by using a calibrated halogen lamp with specified spectral irradiance. The quasi-Fermi level splitting (QFLS_EL_) was calculated according to ${{{\mathrm{QFLS}}}}_{{{{\mathrm{EL}}}}} = k_{{{\mathrm{B}}}}T \times {{{\mathrm{ln}}}}({{{{\mathrm{EQE}}}}_{{{{\mathrm{EL}}}}} \times \frac{{J_{{{\mathrm{G}}}}}}{{J_{0,{\mathrm{rad}}}}}})$, where *k*_B_*T* is the thermal energy at room temperature, and *J*_0,rad_ and *J*_G_ are the radiative recombination current in the dark and the 1-sun-equivalent (AM1.5G) generation current, respectively. The quantification of the *J*_0,rad_ of both subcells is shown in Supplementary Fig. 15 and Supplementary Table 1.

#### Solar cell characteristics

The tandem solar cells were measured in air under AM1.5G (1 sun) equivalent illumination with a Wavelabs Sinus-70 light-emitting diode (LED) class AAA solar simulator as described in a previous publication by Al-Ashouri et al.^7^. The cells were not preconditioned. For calibration we used a slightly modified calibration routine as described in ref. ^58^. We adjusted the spectrum such that it led to the photogenerated current densities obtained by EQE measurements for both subcells. Thus, for a perovskite-limited cell, we first increased the intensity of the blue light to get a silicon-limited cell. Subsequently, the near-infrared region was adjusted until the *J*_SC_ of the silicon-limited tandem solar cell was equal to the *J*_ph,Si_ (calculated from EQE and AM1.5G spectrum). Finally, the intensity of the blue light was decreased until the tandem solar cell was perovskite-limited again and the *J*_SC_ was equal to the *J*_ph,Pero_. For a silicon-limited cell, the procedure is the opposite. The backside of the cell was contacted with a metal vacuum chuck at 25 °C, whereas the front side was contacted with two gold probes. A black laser-cut aperture mask covered the substrate outside of the active area. The *J*–*V* measurements and MPP tracks were recorded using ahome-built LabVIEW software. The EQE spectra were recorded with a home-built set-up using chopped (79 Hz) monochromatic light from a xenon and helium lamp,each covering a specific part of the spectrum. To measure the EQE of the perovskite subcell, the silicon subcell was saturated using an LED with 850 nm peak emission. To maintain short-circuit conditions, a bias voltage of 0.6 V was applied. The silicon subcell was measured by saturating the perovskite subcell with blue light from an LED (455 nm) and applying a bias voltage of 1.0 V. For statistics on totalized integrated subcell current densities of PSTSCs, perovskite absorbers with bandgaps varying from 1.66 to 1.68 eV were used.

### Simulations

#### 3D optical simulations: JCMwave and Bayesian optimization

The data used for the sensitivity analysis presented in Fig. 3 were generated during a layer-thickness optimization with a Bayesian optimization algorithm^59^, similar to an optimization we presented in previous work^13^. During the optimization the thicknesses of the perovskite and nc-SiO_*x*_:H(n) layers were varied to maximize the function min(*J*_ph,pero_,*J*_ph,Si_), which is a well-suited optimization function for monolithic tandem solar cells, as it directly accounts for current matching^60^. The individual simulations were performed with the FEM solver JCMsuite^61^ for a 300–1,190 nm wavelength range and produced absorption spectra for all the layers of the solar cell stack. To be able to do the FEM simulations, we had to restrict the simulation domain to the perovskite top cell and to assume the silicon to be infinitely thick. To compensate, we corrected the absorption of the silicon layer by assuming perfect light trapping for the silicon wafer according to Tiedje and Yablonovitch^62^. From the absorption spectra, the photocurrent densities were calculated^60^. For example, for reflection the equation is given as

$$J_{{{{\mathrm{ph}}}},R} = - e\mathop {\smallint }\limits_{300\;{{{\mathrm{nm}}}}}^{1,190\;{{{\mathrm{nm}}}}} R\left( \lambda \right) \times \varPhi _{{\mathrm{AM1.5G}}}\left( \lambda \right){{{\mathrm{d}}}}\lambda$$

where *e* is the elementary charge, *R* is the spectral reflectance *R* and *Φ* is the spectral photon flux under the AM1.5G condition^60^. Hence, the current-density equivalents can be regarded as solar-spectrum-weighted reflectance values and their relevance for the solar cell device can immediately be seen. Details on the layer thicknesses, optical material data and optimization results are given in Supplementary Table 2.

#### 1D optical simulations: GenPro4

The optical simulations on the RDBL shown in Fig. 5c were performed with GenPro4 developed at TU Delft^63^, which uses the net-radiation method and ray tracing for scattering at the pyramidal back side of the solar cell. Details on the layer stack used for these simulations are given in Supplementary Table 3.

#### Optoelectrical simulations: Quokka

For the RDBL with local contacts, the sheet resistance of the TCO (*R*_sheet_), which depends on its electrical properties and thickness, and the resistance of the grid fingers, which depends on their pitch, geometry and resistivity, have to be optimized. To calculate this balance, electrical simulations were carried out with the Quokka3 software^64^ for which we assumed the wafer properties, finger geometry and resistivity, and Si/TCO/Ag contact resistivities to remain constant. Note also that *R*_sheet_ does not increase linearly with thickness reduction since we considered the variation of its electrical properties in real-solar-cell-like structures as studied in ref. ^27^. The reference point from which the optical power loss (*P*_loss_) is calculated is *J*_SC_ = 19.73 mA cm^−2^ at *t*_TCO_ = 10 nm and FP = 2.5 mm, decreasing to 19.44 mA cm^−2^ at *t*_TCO_ = 200 nm and FP = 0.5 mm. For this *J*_SC_ range variation, *V*_OC_ = 1.9 V and FF = 79.52% are assumed to remain constant. For the electrical *P*_loss_, the holes, vertical, lateral and metal finger transport losses are calculated assuming *J*_SC_ has a constant value of 19.4 mA cm^−2^. Other parameters used can be found in Supplementary Table 4.

## Online content

Any methods, additional references, Nature Research reporting summaries, source data, extended data, supplementary information, acknowledgements, peer review information; details of author contributions and competing interests; and statements of data and code availability are available at 10.1038/s41565-022-01228-8.

## Supplementary information

Supplementary Information Supplementary Figs. 1–15 and Tables 1–4.

## Data Availability

All the data used to plot the figures are available via 10.5442/ND000009 (ref. ^65^). Source data are provided with this paper.

## Source data

Source Data Fig. 1 Source data of Fig. 1d (*AFM*).
Source Data Fig. 2 Source data of Fig. 2c (*fabrication yield*), 2f (*SEM*) and 2g (*GIWAXS*).
Source Data Fig. 3 Source data of Fig. 3a (*EQE, 1-R*), 3b (*Statistical J*_*R*_), 3c (*Statistical J*_*sum*_). Source data for Figs. 3e and 3f (*simulation results sensitivity analysis*), separated as nanotextured and planar data + README for source data of Fig. 3e and 3f.
Source Data Fig. 4 Source data of Fig. 4a (*JV*), 4b (*statistical V*_*OC*_) and 4c (*EL*). Source data of inset in Fig. 4c.
Source Data Fig. 5 Source data of Fig. 5c (*GenPro simulation*), 5d (*Quokka simulation*), 5e (*EQE*) and 5f (*JV*).

## References

[1] EPFL. New world records: perovskite-on-silicon-tandem solar cells (2022); https://actu.epfl.ch/news/new-world-records-perovskite-on-silicon-tandem-sol/

[2] Richter A, Hermle M, Glunz SW (2013). Reassessment of the limiting efficiency for crystalline silicon solar cells. IEEE J. Photovolt..

[3] Bush KA (2018). Compositional engineering for efficient wide band gap perovskites with improved stability to photoinduced phase segregation. ACS Energy Lett..

[4] Bush KA (2018). Minimizing current and voltage losses to reach 25% efficient monolithic two-terminal perovskite–silicon tandem solar cells. ACS Energy Lett..

[5] Mazzarella L (2019). Infrared light management using a nanocrystalline silicon oxide interlayer in monolithic perovskite/silicon heterojunction tandem solar cells with efficiency above 25%. Adv. Energy Mater..

[6] Köhnen E (2019). Highly efficient monolithic perovskite silicon tandem solar cells: analyzing the influence of current mismatch on device performance. Sustain. Energy Fuels.

[7] Al-Ashouri A (2020). Monolithic perovskite/silicon tandem solar cell with >29% efficiency by enhanced hole extraction. Science.

[8] Kim D (2020). Efficient, stable silicon tandem cells enabled by anion-engineered wide-bandgap perovskites. Science.

[9] Isikgor FH (2021). Concurrent cationic and anionic perovskite defect passivation enables 27.4% perovskite/silicon tandems with suppression of halide segregation. Joule.

[10] Xu J (2020). Triple-halide wide–band gap perovskites with suppressed phase segregation for efficient tandems. Science.

[11] Schulze PSC (2020). 25.1% high‐efficiency monolithic perovskite silicon tandem solar cell with a high bandgap perovskite absorber. Sol. RRL.

[12] Santbergen R (2016). Minimizing optical losses in monolithic perovskite/c-Si tandem solar cells with a flat top cell. Opt. Express.

[13] Jäger K, Sutter J, Hammerschmidt M, Schneider P-I, Becker C (2020). Prospects of light management in perovskite/silicon tandem solar cells. Nanophotonics.

[14] Yoo JJ (2021). Efficient perovskite solar cells via improved carrier management. Nature.

[15] Sahli F (2018). Fully textured monolithic perovskite/silicon tandem solar cells with 25.2% power conversion efficiency. Nat. Mater..

[16] Tennyson EM (2021). Multimodal microscale imaging of textured perovskite–silicon tandem solar cells. ACS Energy Lett..

[17] Roß M (2021). Co-evaporated formamidinium lead iodide based perovskites with 1000 h constant stability for fully textured monolithic perovskite/silicon tandem solar cells. Adv. Energy Mater..

[18] Li Y (2021). Wide bandgap interface layer induced stabilized perovskite/silicon tandem solar cells with stability over ten thousand hours. Adv. Energy Mater..

[19] Subbiah AS (2020). High-performance perovskite single-junction and textured perovskite/silicon tandem solar cells via slot-die-coating. ACS Energy Lett..

[20] Chen B (2020). Blade-coated perovskites on textured silicon for 26%-efficient monolithic perovskite/silicon tandem solar cells. Joule.

[21] Hou Y (2020). Efficient tandem solar cells with solution-processed perovskite on textured crystalline silicon. Science.

[22] Zhumagali S (2021). Linked nickel oxide/perovskite interface passivation for high-performance textured monolithic tandem solar cells. Adv. Energy Mater..

[23] Santbergen R (2022). Ray-optics study of gentle non-conformal texture morphologies for perovskite/silicon tandems. Opt. Express.

[24] Chen D (2018). Nanophotonic light management for perovskite–silicon tandem solar cells. J. Photonics Energy.

[25] Tockhorn P (2020). Improved quantum efficiency by advanced light management in nanotextured solution-processed perovskite solar cells. ACS Photonics.

[26] Sutter J (2020). Tailored nanostructures for light management in silicon heterojunction solar cells. Sol. RRL.

[27] Cruz A (2022). Optoelectrical analysis of TCO + silicon oxide double layers at the front and rear side of silicon heterojunction solar cells. Sol. Energy Mater. Sol. Cells.

[28] Bhushan B, Jung YC, Koch K (2009). Micro-, nano- and hierarchical structures for superhydrophobicity, self-cleaning and low adhesion. Philos. Trans. R. Soc. A.

[29] Joanny JF, de Gennes PG (1984). A model for contact angle hysteresis. J. Chem. Phys..

[30] Tadmor R (2021). Open problems in wetting phenomena: pinning retention forces. Langmuir.

[31] Wu J, Xia J, Lei W, Wang B (2013). Advanced understanding of stickiness on superhydrophobic surfaces. Sci. Rep..

[32] Minemawari H (2011). Inkjet printing of single-crystal films. Nature.

[33] Zheng G (2018). Manipulation of facet orientation in hybrid perovskite polycrystalline films by cation cascade. Nat. Commun..

[34] Chen AZ (2017). Crystallographic orientation propagation in metal halide perovskite thin films. J. Mater. Chem. A.

[35] Xi J (2021). Scalable, template driven formation of highly crystalline lead‐tin halide perovskite films. Adv. Funct. Mater..

[36] Luo C (2022). Facet orientation tailoring via 2D-seed-induced growth enables highly efficient and stable perovskite solar cells. Joule.

[37] Kim W (2017). Oriented grains with preferred low-angle grain boundaries in halide perovskite films by pressure-induced crystallization. Adv. Energy Mater..

[38] Chen Q (2014). Controllable self-induced passivation of hybrid lead iodide perovskites toward high performance solar cells. Nano Lett..

[39] Stolterfoht M (2020). How to quantify the efficiency potential of neat perovskite films: perovskite semiconductors with an implied efficiency exceeding 28%. Adv. Mater..

[40] Cho C (2021). Effects of photon recycling and scattering in high-performance perovskite solar cells. Sci. Adv..

[41] Tan WL, McNeill CR (2022). X-ray diffraction of photovoltaic perovskites: principles and applications. Appl. Phys. Rev..

[42] Kim DH (2017). 300% enhancement of carrier mobility in uniaxial-oriented perovskite films formed by topotactic-oriented attachment. Adv. Mater..

[43] Giesbrecht N (2016). Synthesis of perfectly oriented and micrometer-sized MAPbBr_3_ perovskite crystals for thin-film photovoltaic applications. ACS Energy Lett..

[44] Muscarella LA (2019). Crystal orientation and grain size: do they determine optoelectronic properties of MAPbI_3_ perovskite?. J. Phys. Chem. Lett..

[45] Stolterfoht M (2018). Visualization and suppression of interfacial recombination for high-efficiency large-area pin perovskite solar cells. Nat. Energy.

[46] Kirchartz T, Staub F, Rau U (2016). Impact of photon recycling on the open-circuit voltage of metal halide perovskite solar cells. ACS Energy Lett..

[47] Holman ZC, Descoeudres A, Wolf SD, Ballif C (2013). Record infrared internal quantum efficiency in silicon heterojunction solar cells with dielectric/metal rear reflectors. IEEE J. Photovolt..

[48] Boccard M (2017). Low-refractive-index nanoparticle interlayers to reduce parasitic absorption in metallic rear reflectors of solar cells. Phys. Status Solidi A.

[49] Bush KA (2017). 23.6%-efficient monolithic perovskite/silicon tandem solar cells with improved stability. Nat. Energy.

[50] Peña-Camargo F (2020). Halide segregation versus interfacial recombination in bromide-rich wide-gap perovskite solar cells. ACS Energy Lett..

[51] Wolf AJ (2012). Origination of nano- and microstructures on large areas by interference lithography. Microelectron. Eng..

[52] Kern W (1990). The evolution of silicon wafer cleaning technology. J. Electrochem. Soc..

[53] Saliba M (2016). Cesium-containing triple cation perovskite solar cells: improved stability, reproducibility and high efficiency. Energy Environ. Sci..

[54] Pierce E, Carmona FJ, Amirfazli A (2008). Understanding of sliding and contact angle results in tilted plate experiments. Colloids Surf. A.

[55] Zizak I (2016). The mySpot beamline at BESSY II. J. Large-Scale Res. Facil..

[56] Benecke G (2014). A customizable software for fast reduction and analysis of large X-ray scattering data sets: applications of the new DPDAK package to small-angle X-ray scattering and grazing-incidence small-angle X-ray scattering. J. Appl. Crystallogr..

[57] Jiang Z (2015). GIXSGUI: a MATLAB toolbox for grazing-incidence X-ray scattering data visualization and reduction, and indexing of buried three-dimensional periodic nanostructured films. J. Appl. Crystallogr..

[58] Meusel M, Adelhelm R, Dimroth F, Bett AW, Warta W (2002). Spectral mismatch correction and spectrometric characterization of monolithic III–V multi-junction solar cells. Prog. Photovolt: Res. Appl..

[59] Schneider, P.-I., Garcia Santiago, X., Rockstuhl, C. & Burger, S. Global optimization of complex optical structures using Bayesian optimization based on Gaussian processes. In *Digital Optical Technology 2017* (eds Kress B. C. et al.) 103350O (SPIE, 2017); 10.1117/12.2270609

[60] Jäger K, Korte L, Rech B, Albrecht S (2017). Numerical optical optimization of monolithic planar perovskite–silicon tandem solar cells with regular and inverted device architectures. Opt. Express.

[61] Pomplun J, Burger S, Zschiedrich L, Schmidt F (2007). Adaptive finite element method for simulation of optical nano structures. Physica Status Solidi B Basic Solid State Phys..

[62] Tiedje T, Yablonovitch E, Cody GD, Brooks BG (1984). Limiting efficiency of silicon solar cells. IEEE Trans. Electron Devices.

[63] Santbergen R (2017). GenPro4 optical model for solar cell simulation and its application to multijunction solar cells. IEEE J. Photovolt..

[64] Fell A (2013). A free and fast three-dimensional/two-dimensional solar cell simulator featuring conductive boundary and quasi-neutrality approximations. IEEE Trans. Electron Devices.

[65] Tockhorn, P. et al. Supplement to: Nano-optical designs for high efficiency monolithic perovskite–silicon tandem solar cells (HZB Data Service, 2022); 10.5442/ND000009 10.1038/s41565-022-01228-8 PMC9646483 36280763

