# Supplementary material for: Nano-optical designs for high-efficiency monolithic perovskite–silicon tandem solar cells
Source: Nat Nanotechnol. 2022 Oct 24;17(11):1214–21. doi: 10.1038/s41565-022-01228-8 (PMC9646483; doi:10.1038/s41565-022-01228-8)
Supplement: Supplementary file 1 — Supplementary Figs. 1–15 and Tables 1–4. [file 41565_2022_1228_MOESM1_ESM.pdf]

nature nanotechnology

Article

https://doi.org/10.1038/s41565-022-01228-8

Nano-optical designs for high-efficiency monolithic perovskite–silicon tandem solar cells

In the format provided by the authors and unedited

Supporting Information

Nano-optical designs for high efficiency monolithic perovskite-silicon tandem solar cells

Philipp Tockhorn[1#], Johannes Sutter[1#], Alexandros Cruz[1], Philipp Wagner[1], Klaus Jäger[1,2], Danbi Yoo[1], Felix Lang[3], Max Grischek[1,3], Bor Li[1], Jinzhao Li[1], Oleksandra Shargaieva[1], Eva Unger[1,4], Amran Al-Ashouri[1], Eike Köhnen[1], Martin Stolterfoht[3], Dieter Neher[3], Rutger Schlatmann[1,5], Bernd Rech[1,6], Bernd Stannowski[1,7], Steve Albrecht[1,6], Christiane Becker[1,5]**

[1] Helmholtz-Zentrum Berlin für Materialien und Energie GmbH, Division Solar Energy, Kekuléstr. 5, 12489, Berlin, Germany

[2] Zuse Institute Berlin, Computational Nanooptics Group, Takustr. 7, 14195 Berlin, Germany

[3] Universität Potsdam, Soft matter physics, Karl-Liebknecht-Straße 24/25, 14476 Potsdam, Germany

[4] Humboldt Universität zu Berlin, Department of Chemistry, Brook-Taylor-Strasse 2, 12489 Berlin, Germany

[5] Hochschule für Technik und Wirtschaft Berlin, Faculty 1: School of Engineering – Energy and Information, 10313 Berlin, Germany

[6] Technische Universität Berlin, Faculty of Electrical Engineering and Computer Science, 10587 Berlin, Germany

[7] Berliner Hochschule für Technik, 13353 Berlin

# These authors contributed equally to this work. Order determined by cast of 298 sided digital dice.

* Email: steve.albrecht@helmholtz-berlin.de, christiane.becker@helmholtz-berlin.de

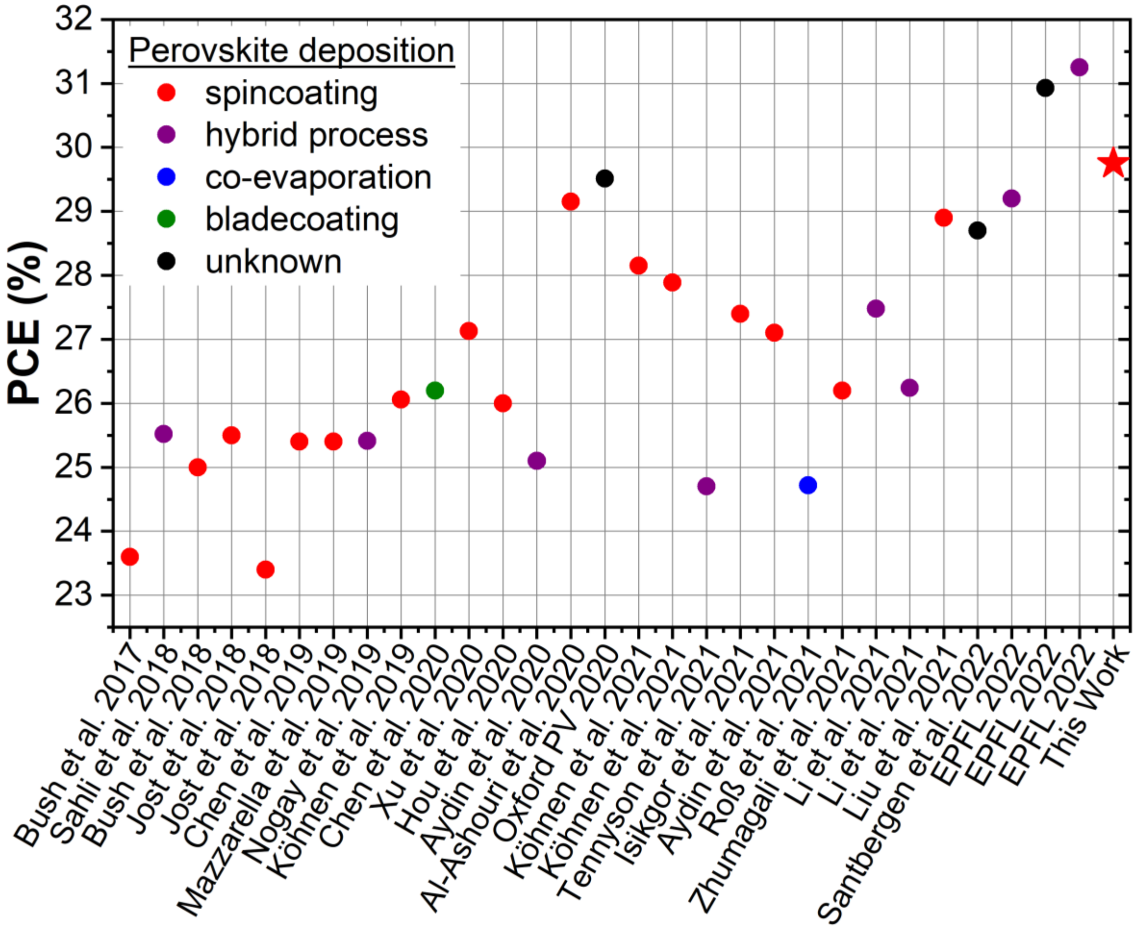

Figure S1 | Overview of 2-terminal perovskite/silicon tandem solar cells (PSTSC) with power conversion efficiency (PCE) >23%. The different colors mark the deposition technique of the perovskite absorber, as displayed in the legend.

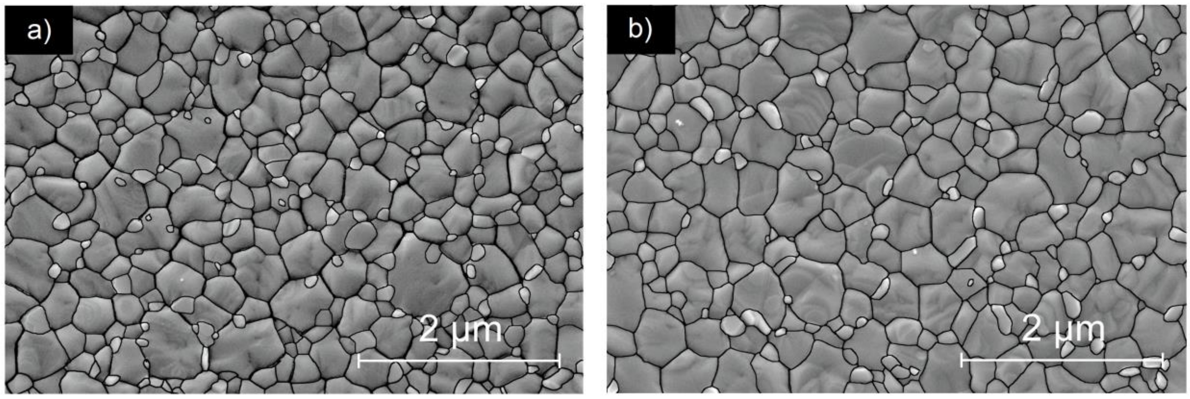

Figure S2 | Top-view scanning electron microscope (SEM) images of bare perovskite layers deposited on planar (a) and nanotextured (b) substrates. The grain size analysis in Fig. 2f is based on these images.

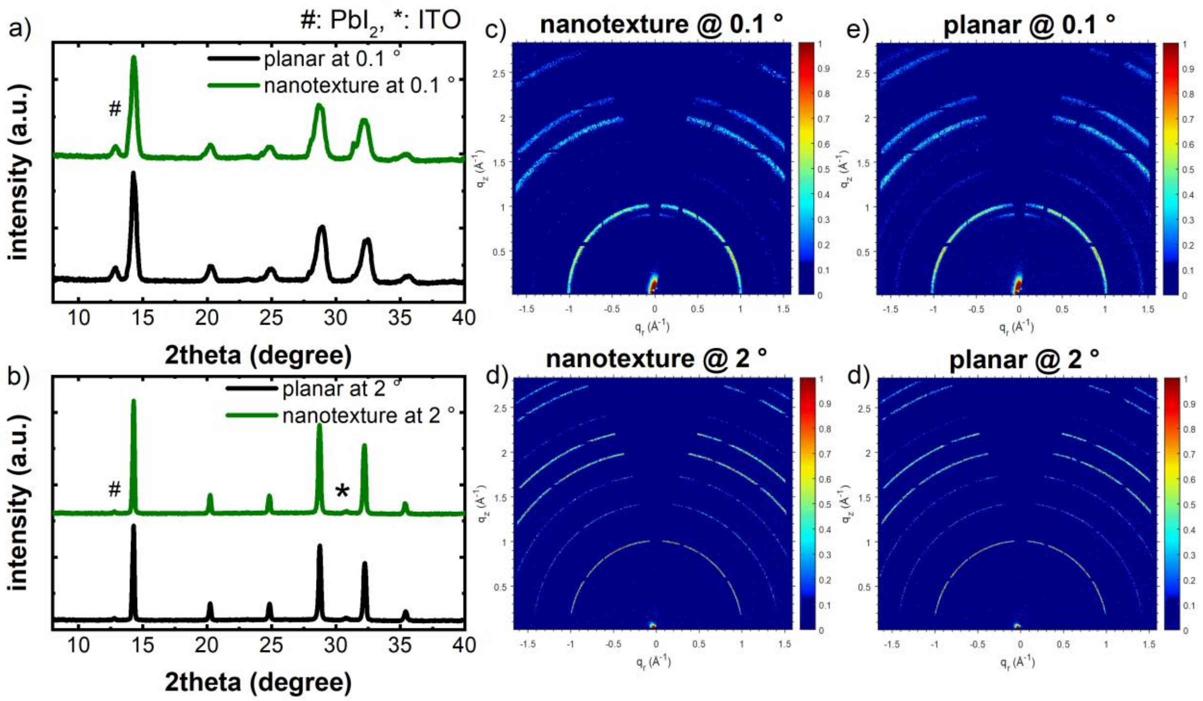

Figure S3 | (a-b) Azimuthally integrated diffraction patterns and (c-f) GIWAXS maps at grazing incidence angles of 0.1 and 2° of perovskite layers deposited onto planar (a,c,e) and nanotextured (b,d,f) silicon bottom cells.

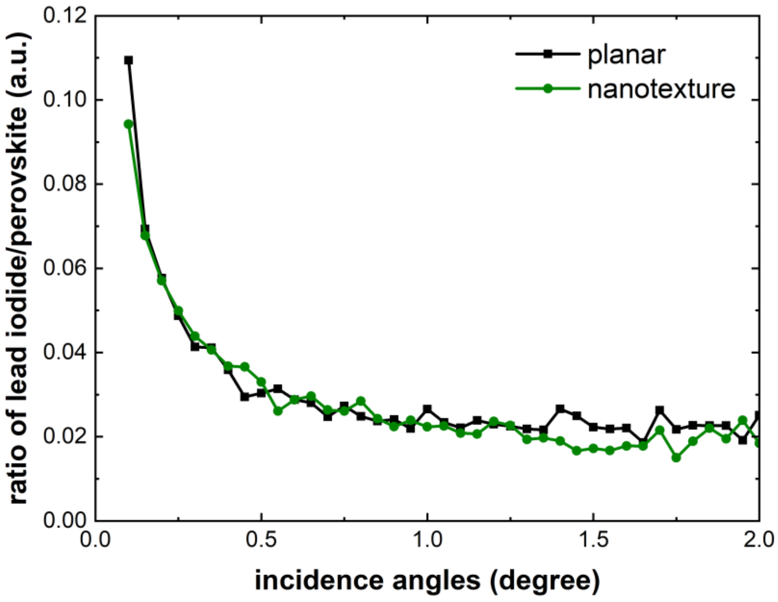

Figure S4 | Integrated peak area ratio of (001) reflection of lead iodide/ (100) reflection of perovskite versus incidence angles. Considering that the probing depth increases with incidence angle, the results indicate the accumulation of Pb on the surface.

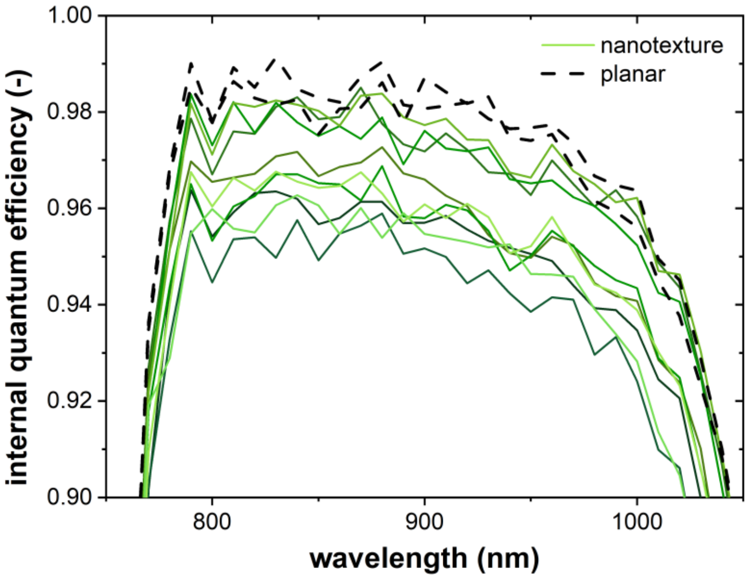

Figure S5 | Internal quantum efficiency (IQE) defined as IQE = EQE/(1-*R*) of planar (black dashed lines) and nanotextured silicon bottom cells (green lines).

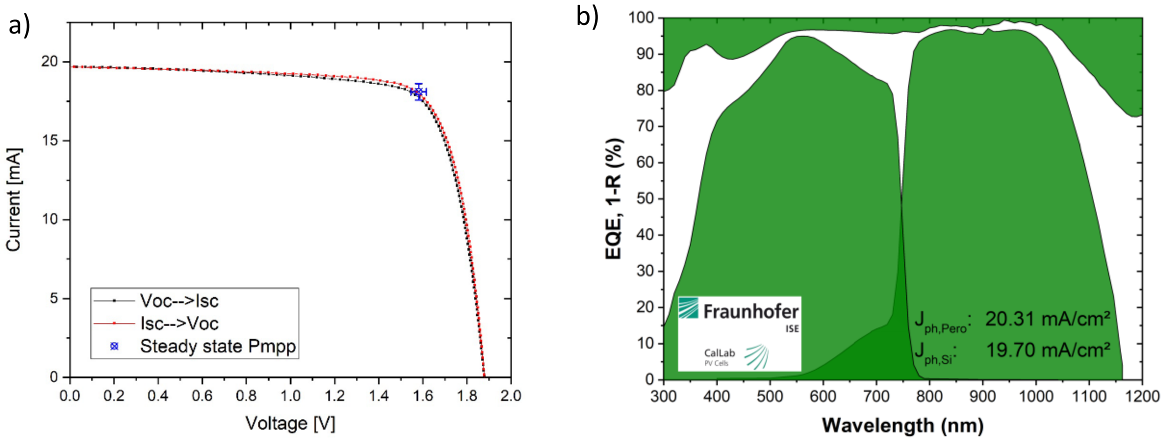

Figure S6 | (a) Current/voltage (*J-V*) characteristics of a nanotextured PSTSC with standard rear reflector (no RDBL) certified by CalLab at Fraunhofer ISE. The maximum power point (MPP) value is marked as a blue cross. (b) Corresponding external quantum efficiency (EQE) measurement of a nanotextured PSTSC with standard rear reflector with summed up short circuit current density from EQE ($J_{ph,pero}$+$J_{ph,Si}$) = 40 mA/cm^2 the 1-*R* spectrum was measured in-house. Note that the EQE spectra were measured in the wavelength range from 300-1162 nm, which leads to a slight underestimation of the photogenerated current density of the silicon subcell.

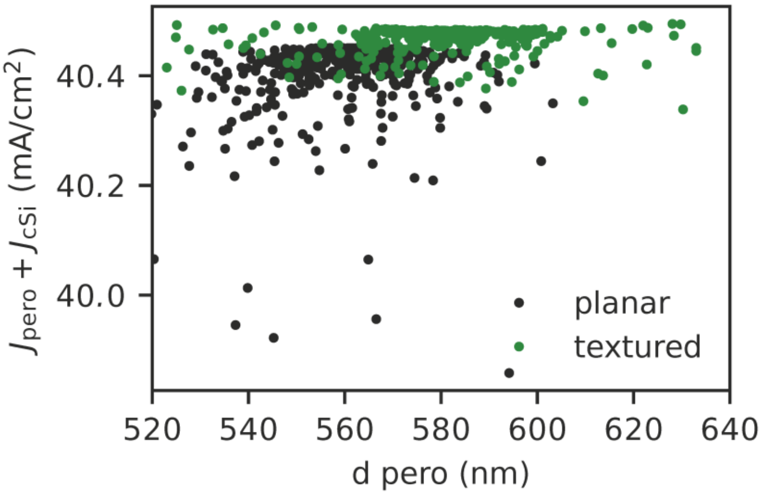

Figure S7 | Combined photogenerated current density (J_{Pero}+J_{Si}) of planar and nanotextured PSTSCs as a function of different perovskite layer thicknesses. The data points were generated with optical simulations using the finite element method. These simulations were part of a sensitivity analysis, which is discussed in the main manuscript and presented in Figs. 3e and 3f.

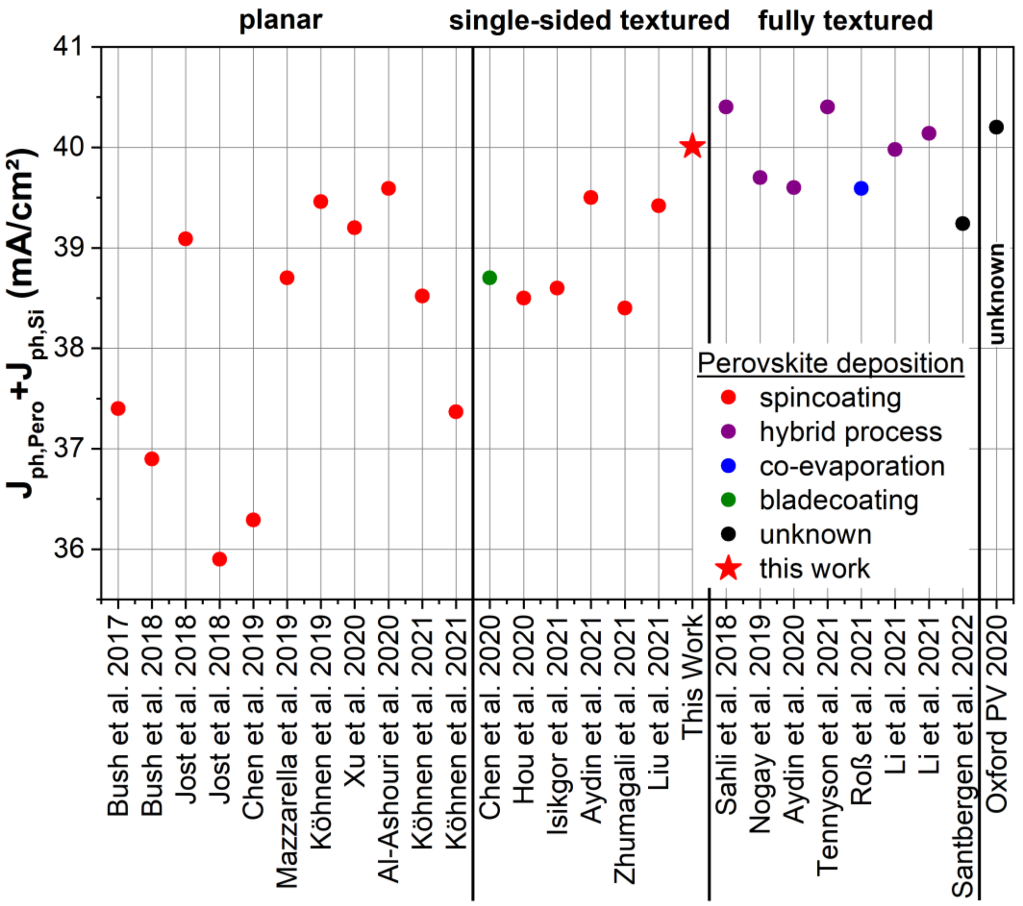

Figure S8 | Combined photogenerated current density of perovskite and silicon sub cell ($J_{ph,Pero}$+$J_{ph,Si}$) for selected publications of highly efficient perovskite/silicon tandem solar cells (PSTSC). The values are taken from the reported EQE-spectra in the respective publications. The graph is structured into PSTSCs with planar, single-sided and fully textured perovskite top cell.

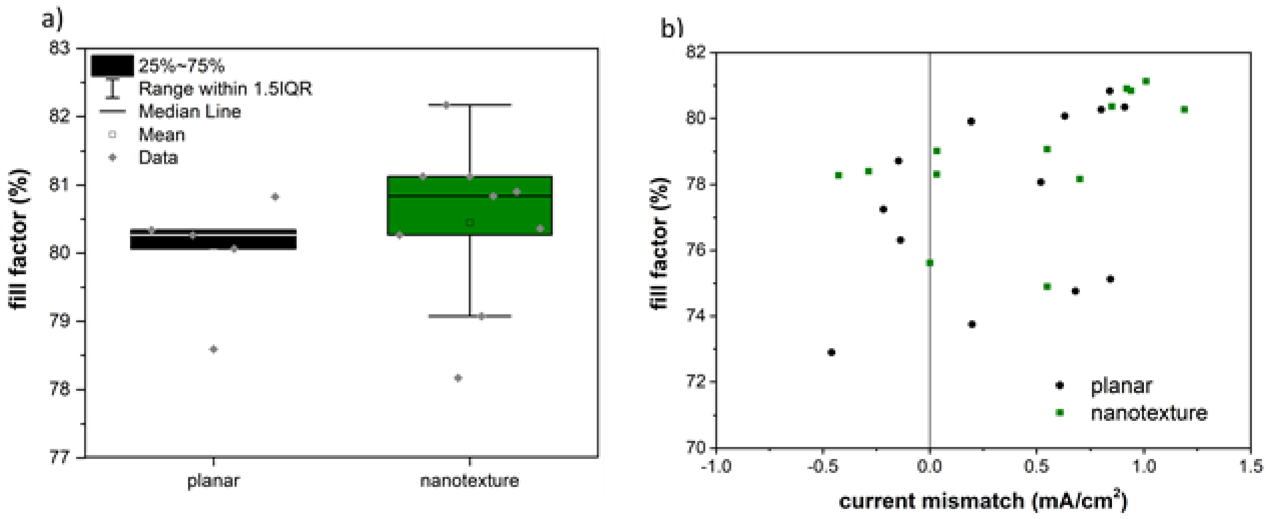

Figure S9 | (a) Boxplots of the fill factor for nanotextured and planar tandem solar cells (one batch). (b) Fill factor *(FF)* of various planar and nanotextured perovskite/silicon tandem solar cells (PSTSC) as a function of current mismatch ($J_{ph,Pero}$ - $J_{ph,Si}$) (various batches with bandgaps of 1.66 eV - 1.68 eV).

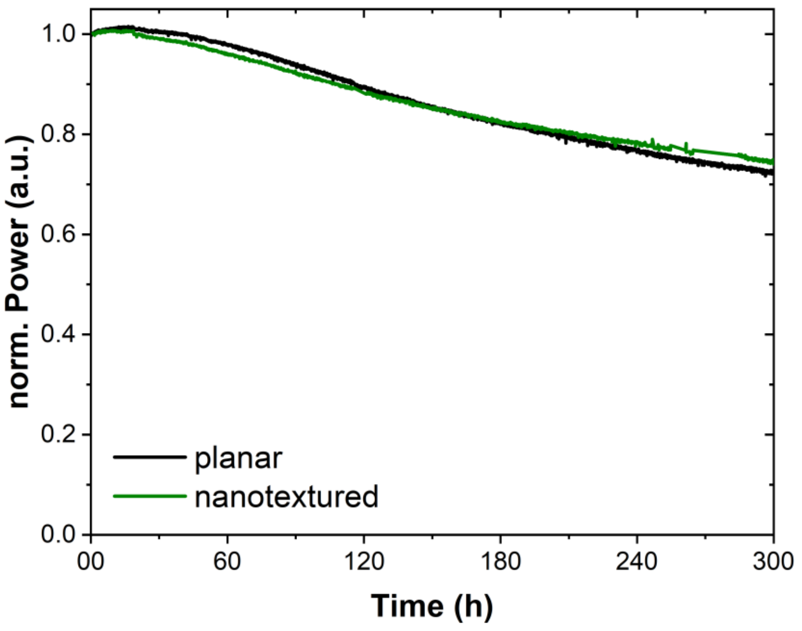

Figure S10 | Long-term maximum power point (MPP) track under a dichromatic LED illumination of non-encapsulated perovskite/silicon tandem solar cells (PSTSC) in air at a controlled temperature of 25°C and relative humidity of 30 to 40%.

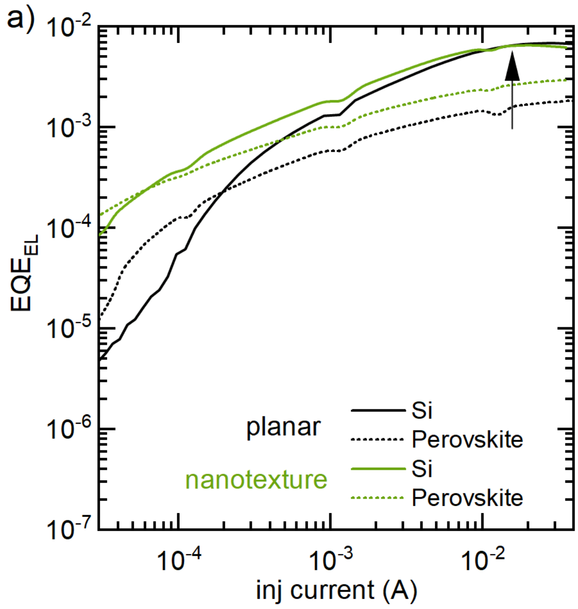

Figure S11 | Electroluminescence quantum yield (EQE_{EL}) of the perovskite and c-Si subcells in planar and nanotextured perovskite/silicon tandem solar cells (PSTSC). Interestingly, the Si EQE_{EL} changes barely, while the Perovskite increases by approximately 60% (relative) for the nanotextured device compared to the planar PSTSC.

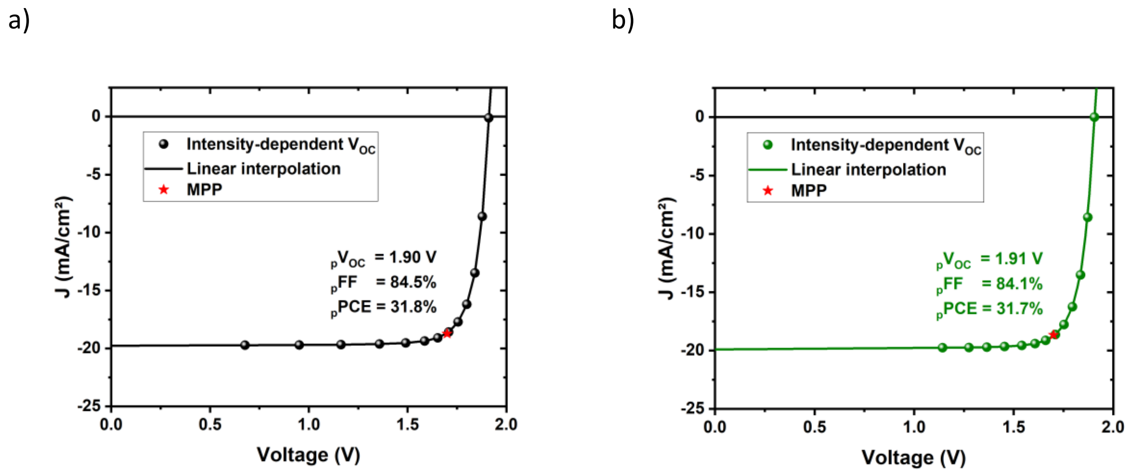

Figure S12 | Pseudo *JV*-curves of a) planar and b) nanotextured PSTSC as determined from intensity-dependent *JV*-curves. The pseudo solar cell parameters are noted in the respective graphs.

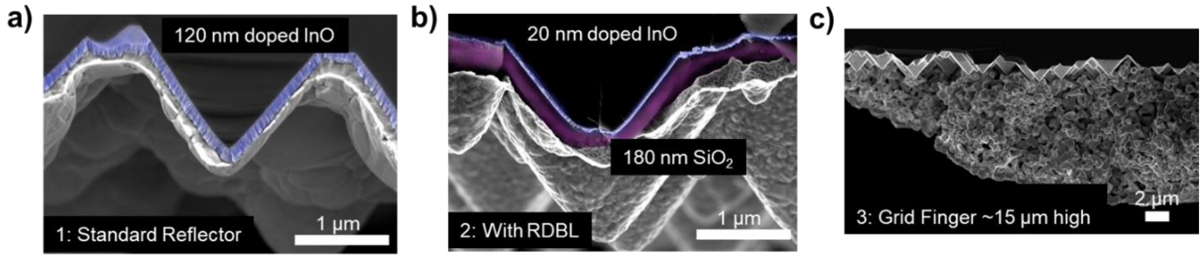

Figure S13 | Cross-sectional scanning electron microscopy (SEM) images of different rear reflectors: 1. Standard reflector, 2. reflector with dielectric buffer layer (RDBL) and 3. Local contacts of the RDBL.

Figure S14 | Certificate of the 29.8% efficient PSTSC

Fraunhofer ISE CalLab PV Cells

Heidenhofstr.2

79110 Freiburg

Fraunhofer ISE

CalLab PV Cells

Werkskalibrierschein
Proprietary calibration report

10005172HMI1020

Gegenstand<br>*Object*	**monofacial solar cell**
Hersteller<br>*Manufacturer*	**Helmholtz**
Typ<br>*Type*	**PSC/Si**
Fabrikat/Serien-Nr.<br>*Serial number*	**HMI005 / 180604**
Auftraggeber<br>*Customer*	**Helmholtz-Center Berlin**<br>**Young Investigator Group Perovskite Tandem Solar Cells**<br>**Kekuléstr. 5**<br>**12489 Berlin**<br>**Germany**
Auftragsnummer<br>*Order No.*	**172HMI1021**
Anzahl der Seiten<br>*Number of pages*	**6**
Datum der Kalibrierung<br>*Date of calibration*	**12.11.2021**

Kalibrierscheine ohne Unterschrift haben keine Gültigkeit. *Calibration certificates without signature are not valid.*

Datum<br>*Date*	Leiter des Kalibrierlaboratoriums<br>*Head of the calibration laboratory*	Bearbeiter<br>*Person in charge*
16.11.2021	*Jochen Hohl-Ebinger*	*Astrid Semeraro*

1. Beschreibung des Kalibriergegenstandes

Description of the calibrated object

Das Messobjekt ist eine Tandem-Solarzelle . Typ: Perowskit/Silicium.
The device under test is a perovskite-silicon tandem solar cell.

2. Messverfahren

Measurement procedure

Die Kalibrierung des Kalibrierobjektes wird gemäß /1/ mit einem Zweilampen-DC-Sonnensimulator durchgeführt. Die Einstrahlung wird mit Hilfe einer Monitorzelle während der gesamten Messdauer aufgenommen und deren Schwankungen bezüglich der Messung korrigiert. Die Divergenz der Randstrahlen ist < 5°. Die Solarzelle wird auf einem Vakuumprobentisch thermisch stabilisiert.
The calibration of the test sample was performed at Standard Testing Conditions (STC) with a dual light steady-state solar simulator according to /1/. The irradiance is controlled with a monitor cell during the measurement in order to correct fluctuations. The divergence of the peripheral beams is < 5°. The solar cell is kept at a constant temperature

Rückführung der Referenzsolarzellen/*Traceability of the reference solar cells* :

Identitäts-Nr. / *Identity-Nr.* :	Kalibrierschein-Nr./ *Certificate-Nr.* :	Rückführung/ *Traceability* :
ISE020008	47056-PTB-20	PTB
ISE021030	47077-PTB-21	PTB

Die Korrektur der spektralen Fehlanpassung (Mismatch), die durch die Abweichung der spektralen Verteilung des Sonnen Simulators vom Standard-Spektrum AM1.5G /3/ in Kombination mit den verschiedenen spektralen Empfindlichkeiten von Referenzzelle und Messobjekt entsteht /4/, wurde durch eine erweiterte Mismatchberechnung /4/ - wie in /2/ beschrieben - korrigiert.
Dazu wurde die spektrale Verteilung der Bestrahlung (Sonnensimulator) mit einem Spektralradiometer und die spektrale Empfindlichkeit des Messobjektes mit einem laserbasierten Messplatz /5/ gemessen (s. Kalibrierschein Nr: 9005172HMI1020).
The spectral mismatch - caused by the deviation of the simulator spectrum from the standard spectrum AM1.5G /3/ in combination with the difference between the spectral response of the reference cell and that of the device under test (DUT) – is calculated by a generalized mismatch correction /3/ as described in /2/.
For the spectral mismatch correction the spectral distribution of the solar simulator is measured with a spectroradiometer, the spectral response of the DUT is measured with a laser-based setup according to /5/ (cf. Calibration Mark: 9005172HMI1020.

Der P_{MPP} wurde durch MPP-Tracking über 300s bestimmt. Der angegebene P_{MPP} ist der Mittelwert von 906-1205s dieser stabilisierten Messung. Anschließend wurde die IV-Kennlinie in zwei Richtungen (V_{OC} -> I_{SC} und I_{SC} -> V_{OC}) aufgenommen.
The P_{MPP} was determined by MPP-Tracking for 300s. The reported P_{MPP} represents the average value of the range 906-1205s of this stabilized measurement. Afterwards, the IV-curve was determined with a scan in both directions (V_{OC} -> I_{SC} and I_{SC} -> V_{OC}).

Fraunhofer ISE

CalLab PV Cells

Die Rückführung der Spektralmessung auf SI-Einheiten erfolgte über den Vergleich mit einer Standardlampe.
The traceability of the measurement of the spectral distribution to SI-Units is achieved using a standard lamp for the calibration of the spectroradiometer.

Identitäts-Nr. / *Identity-Nr.* :	Kalibrierschein-Nr./ *Certificate-Nr.* :	Rückführung/ *Traceability* :
BN-9101-451	40006-20-PTB	PTB

3. Messbedingungen

Measurement conditions

Standardtestbedingungen (STC) / *Standard Testing Conditions (STC)* :

Absolute Bestrahlungsstärke / *Total irradiance* :	1000	W/m²
Nominalwert der Temperatur des Messobjektes / Nominal Value of *Temperature of the DUT* :	25	°C
Spektrale Bestrahlungsstärke / *Spectral irradiance distribution* :	AM1.5G Ed.4 (2019)	

Die Messung der IV-Kennlinie (Strom-Spannungs-Kennlinie) des Messobjektes erfolgt mit Hilfe eines Vierquadranten-Netzteiles und eines Kalibrierwiderstandes. Die Temperatur der Solarzelle wird mit einem Tastsensor ermittelt und auf (25±0,5)°C eingestellt.
The measurement of the IV-curve is performed with a 4-quadrant power amplifier and a calibration resistor. The temperature of the solar cell is determined by a sensor and adjusted to (25±0.5)°C.

4. Messergebnis

Measurement results

Fläche / *Area* (da)[1]: = (1.0163 ± 0.0063) cm²

[1] *: (t) = total area, (ap) = aperture area, (da) = designated illumination area /7/*

Kennlinienparameter des Messobjektes unter Standardtestbedingungen (STC) / *IV-curve parameter under Standard Testing Conditions (STC)* :

		Vorwärtsrichtung / *forwards scan direction*	Rückwärtsrichtung / *reverse scan direction*	steady state MPP
V_{oc}	=	(1919 ± 19) mV	(1919 ± 19) mV	
I_{SC} (Ed.2 - 2008)	=	(19.80 ± 0.38) mA	(19.74 ± 0.38) mA	
I_{MPP}	=	18.26 mA	18.17 mA	(18.30 ± 0.51) mA
V_{MPP}	=	1652 mV	1652 mV	(1655 ± 35) mV
P_{MPP}	=	30.17 mW	30.01 mW	(30.28 ± 0.81) mW
FF	=	79.4 %	79.2 %	
η	=			(29.80 ± 0.82) %

Angegeben ist jeweils die erweiterte Messunsicherheit, die sich aus der Standardmessunsicherheit durch Multiplikation mit dem Faktor *k*=2 ergibt. Sie wurde gemäß dem "Guide to the expression of Uncertainty in Measurement" ermittelt. Sie entspricht bei einer Normalverteilung der Abweichungen vom Messwert einer Überdeckungswahrscheinlichkeit von 95%.
The expanded measurement uncertainty resulting from the standard measurement uncertainty multiplied with a factor k=2 is specified. The calculation was carried out according to the "Guide to the expression of Uncertainty in Measurement". The value corresponds to a Gaussian distribution denoting the deviations of the measurement value within a probability of 95%.

5. Zusatzinformationen

Additional information

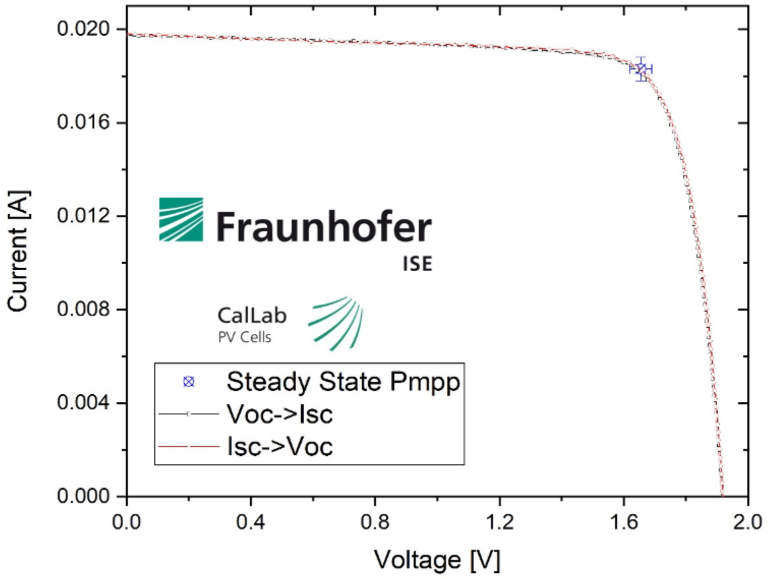

Angegeben ist jeweils die erweiterte Messunsicherheit, die sich aus der Standardmessunsicherheit durch Multiplikation mit dem Faktor *k*=2 ergibt. Sie wurde gemäß dem "Guide to the expression of Uncertainty in Measurement" ermittelt. Sie entspricht bei einer Normalverteilung der Abweichungen vom Messwert einer Überdeckungswahrscheinlichkeit von 95%.
The expanded measurement uncertainty resulting from the standard measurement uncertainty multiplied with a factor k=2 is specified. The calculation was carried out according to the "Guide to the expression of Uncertainty in Measurement". The value corresponds to a Gaussian distribution denoting the deviations of the measurement value within a probability of 95%.

5. Zusatzinformationen

Additional information

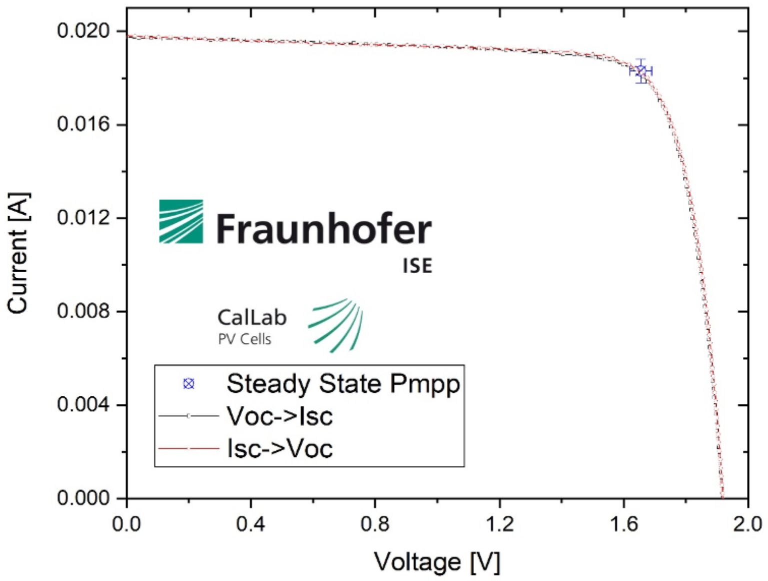

Fraunhofer ISE

CalLab PV Cells

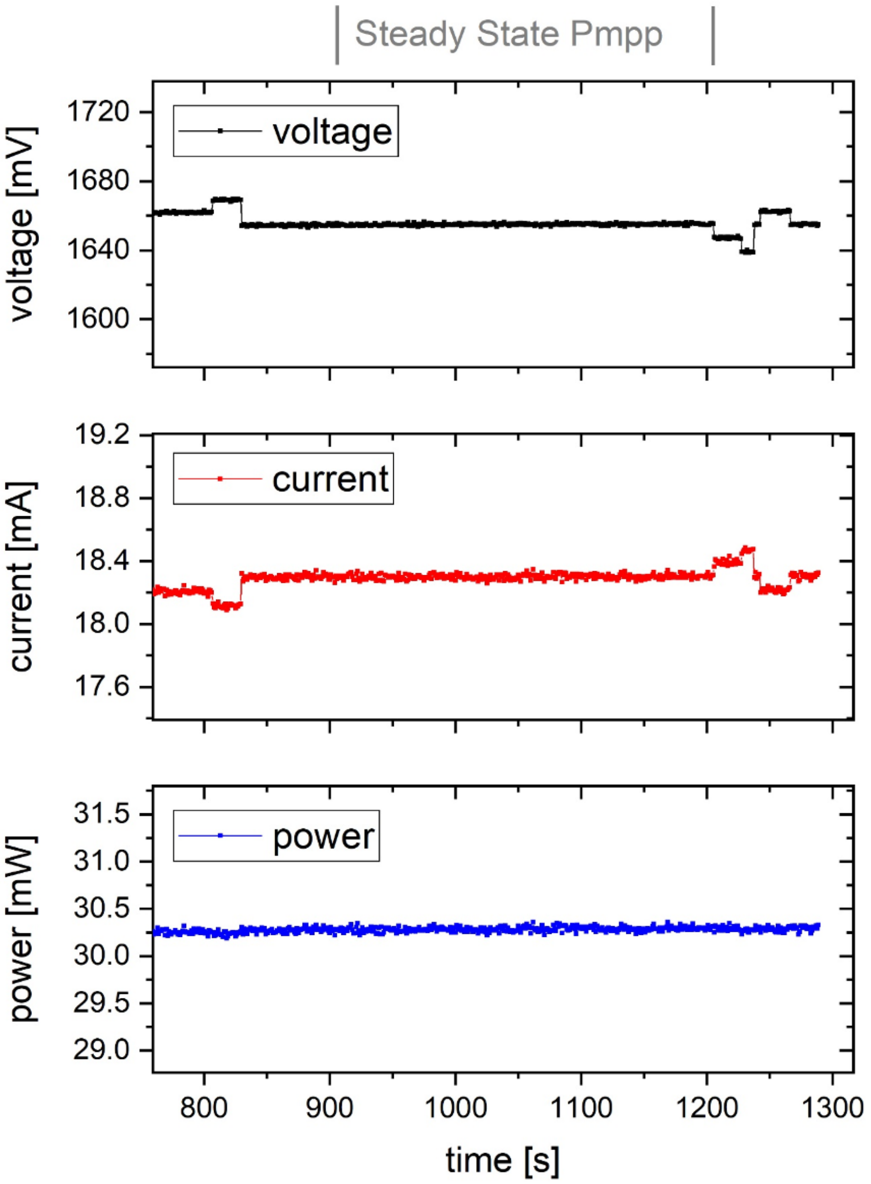

Fraunhofer ISE

CalLab PV Cells

6.Literatur

Literature

/1/ IEC 60904-1-Ed.3:2020, *Photovoltaic devices - Part 1: Measurement of photovoltaic current-voltage characteristics*

/2/ M. Meusel, R. Adelhelm, F. Dimroth, A.W. Bett, W. Warta Spectral Mismatch Correction and Spectrometric Characterization of Monolithic III–V Multi-junction Solar Cells Prog. Photovolt: Res. Appl. 10 (2002) p. 243–255

/3/ IEC 60904-3-Ed.4:2019, *Photovoltaic devices - Part 3: Measurement principles for terrestrial photovoltaic (PV) solar devices with reference spectral irradiance data*

/4/ IEC 60904-7-Ed.4:2019, *Photovoltaic devices - Part 7: Computation of the spectral mismatch error introduced in the testing of a photovoltaic device*

/5/ IEC 60904-8-Ed.3:2014*, Photovoltaic devices - Part 8: Measurement of the spectral responsivity of a photovoltaic (PV) device*

/6/ IEC 60904-9-Ed.3:2020, *Photovoltaic devices - Part 9: Solar simulator performance requirements*

/7/ M.A. Green, K. Emery, Y. Hishikawa, W. Warta, and E. D. Dunlop, *Solar cell efficiency tables (version 39)*. Progress in Photovoltaics: Research and Applications, 2012. 20: p. 12-20.

Hinweis: Es ist nicht gestattet, ohne die schriftliche Genehmigung des ISE CalLab PV Cells den Werkskalibrierschein auszugsweise zu vervielfältigen.
Note: *This proprietary calibration report may not be reproduced other than in full. Extracts may be taken only by the written permission of ISE CalLab PV Cells.*

Ende des Kalibrierscheins / End of certificate

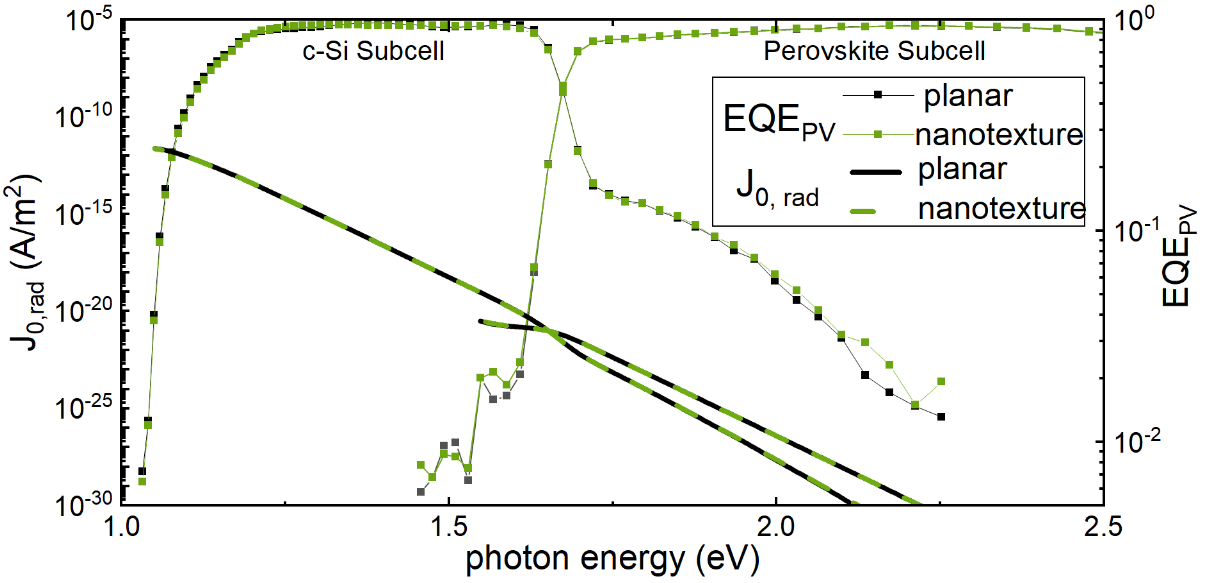

Figure S15 | External quantum efficiency (EQE_{PV}) onset of the silicon and perovskite subcells and their emitted spectral photon flux calculated when the device is in equilibrium with the black-body (BB) radiation of the surroundings at 300K according to equation S1 and S2.

$$J_{0,\mathrm{rad}} = \int \mathrm{EQE}\ \phi_{\mathrm{BB}}\ d\epsilon \qquad \text{(eq. S1)}$$

with

$$\phi_{\mathrm{BB}} = \frac{1}{4\hbar^3 c^2} \cdot \frac{E^2}{\exp\left(\frac{E}{k_B T}\right) - 1} \qquad \text{(eq. S2)}$$

Table S1 | Summary of the derived $J_{0,rad}$ values for the respective perovskite top and silicon bottom cells

	$J_{0,\mathrm{rad}}$ **perovskite subcell** (A/m^2)	$J_{0,\mathrm{rad}}$ **silicon subcell** (A/m^2)
planar	3.14x10^{-21}	2.39x10^{-12}
nanotexture	3.42 x10^{-21}	2.28 x10^{-12}

Table S2 | Details of the 3D optical simulations with the finite element method (FEM)

The materials and layer thicknesses used for the optical simulations presented in Figures 3d-f. All thicknesses (except Si) are given in nm, the bold thicknesses resulted from the Bayesian optimization. The references for refractive index data are given in the second column. Further, the optimized photo-current densities are shown.

		**Planar**	**Nanotextured**
Lithium fluoride (LiF)	[S1]	110	110
Indium zinc oxide (IZO)	[S2]	90	90
Tin oxide (SnO_2)	[S2]	10	10
C_{60}	[S3]	23	23
Perovskite[1]	[S3]	**563**	**583**[2]
Indium tin oxide (ITO)	[S3]	21	21
Nanocrystalline silicon oxide (nc-SiO_x)	[S4]	**101**	**110**
Intrinsic amorphous hydrogenated Si (a-Si:H)	[S5]	5	5
Crystalline silicon (wafer, Si)	[S6]	300 µm	300 µm
$J_{ph,max}$ (mA/cm^2)		20.22	20.24

1 As we did not have optical data for the perovskite used in this work, we performed the simulations with optical data for the triple-cation perovskite $Cs_{0.05}(MA_{0.17}FA_{0.83}) Pb(I_{0.83}Br_{0.17})_3$ with ≈ 1.64 eV bandgap instead. The self-assembled monolayer (SAM) between ITO and perovskite can be omitted in the optical simulations due to its negligible thickness.

2 For the nanotextured device, the perovskite layer is not conformal. The thickness denotes the thickness of a planar layer with the same volume.

Table S3 | Details of the 1D optical simulations with `GenPro4`

The materials and layer thicknesses used for the optical simulations considering the solar cell layer stack shown in Figure 5. All thicknesses (except Si) are given in nm. The references for refractive index data are given in the second column.

		Planar
Lithium fluoride (LiF)	[S1]	110 nm
Indium zinc oxide (IZO)	[S2]	90 nm
Tin oxide (SnO_2)	[S2]	10 nm
C_{60}	[S3]	23 nm
perovskite[1]	[S3]	569 nm
Doped indium oxide (InO) interlayer	[S7]	20 nm
Nanocrystalline silicon oxide (nc-SiO_x)	[S4]	107 nm
Intrinsic amorphous hydrogenated Si (a-Si:H)	[S8]	5 nm
Crystalline silicon (wafer, Si)	[S5]	260 µm
p-doped amorphous hydrogenated Si (p-a:Si:H)	[S4]	10 nm
Doped indium oxide (InO) rear-side	[S7]	10-200 nm<br>10nm Steps
Silicon oxide (SiO_2)	[S7]	0-300 nm<br>10 nm Steps
Silver (Ag)	[S9]	400 nm

Table S4 | Quokka3 simulation parameters

Parameter	Value, Property, Model, Variation	Unit
Dimensions	2	
Unit Cell Thickness	260	µm
Unit Cell width	20000	µm
<u>Material Group</u>		
Optical Generation Model Type	Text-Z model obtained from GenPro4 Simulation	
Auger Model	Si-Altermatt2011	
Radiative Recombination Model	Si-Nguyen	
Density of State	Si-Green1990	
Intrinsic Carrier Density Model	user-const	
Intrinsic Carier Density n_{i0}	9.65e9	cm^{-3}
Silicon Mobiylity Model	Si-Klaassen1992	
Si Band Gap Narrowing Model	Si-Schenk1998	
Doping Setting Type	dopingtype-resistivity	
Doping Type	n-type	
Doping Resistivity	3	Ωcm
Bulk Recombination Type	intrinsic plus SRH	
Bulk Recombination SRH Type	τ-E_t	
Bulk Recombination SRH τ_n	1000	µs
Bulk Recombination SRH τ_p	10000	µs
Bulk Recombination SRH τ E_t-E_i	0	eV
<u>Electron Selective Contact</u>		
Geometry Plane	front	
Geometry Shape	full	
Electrical Model Type	lumped	
Sheet Resistance (R_{sh})	0.001	Ω
Conduction Type	n-type	
Vertical Resistivity Type	ohmic	
Vertical Resistivity	0.05	Ωcm^2
Contact Recombination Type	Surface effective Recombination (S_{eff})	
Contact Recombination S_{eff}	1.2	cm/s
**Electron Contact Feature**		
Geometry Plane	front	
Geometry Shape	full	
Ohmic Resistivity (TCO/Metal)	3e-4	Ωcm^2
Current Transport Model	ohmic	
**Electron Metal Feature**		
Geometry Plane	front	
Geometry Shape	full	
Polarity	n-type	
Sheet Resistance (R_{sh})	1.5e-3	Ω
Shading Fraction	0	
<u>Hole Selective Contact</u>		

Geometry Plane	rear	
Geometry Shape	full	
Electrical Model Type	lumped	
Sheet Resistance (R_{sh})	95–216	Ω
Conduction Type	p-type	
Vertical Resistivity Type	ohmic	
Vertical Resistivity	0.2	Ωcm²
Contact Recombination Type	Surface effective Recombination (S_{eff})	
Contact Recombination S_{eff}	1.2	cm/s
**Hole Contact Feature**		
Geometry Plane	rear	
Geometry Shape	rectangle	
Geometry Size X (width)	40	µm
Geometry Pitch X (finger pitch)	500–2500	µm
Ohmic Resistivity (TCO/Metal)	1e–5	Ωcm²
Current Transport Model	ohmic	
**Hole Metal Feature**		
Geometry Plane	rear	
Geometry Shape	rectangle	
Geometry Size X (width)	40	µm
Geometry Pitch X (finger pitch)	500–2500	µm
Polarity	p-type	
Sheet Resistance (R_{sh})	1.5e–3	Ω
Shading Fraction	0	

[S1] H. H. Li, ‘‘Refractive index of alkali halides and its wavelength and temperature derivatives,’’ J. Phys. Chem. Ref. Data, vol. 5, no. 2, pp. 329–528, 1976. doi.org/10.1063/1.555536

[S2] M. Jošt, E. Köhnen, A. B. Morales-Vilches, et al., ‘‘Textured interfaces in monolithic perovskite/silicon tandemsolar cells: advanced light management for improved efficiency and energy yield,’’ Energy Environ. Sci., vol. 11, no. 12, pp. 3511–3523, 2018. doi.org/10.1039/C8EE02469C

[S3] A. Tejada Esteves, Personal Communication. 2020

[S4] L. Mazzarella, M. Werth, K. Jäger, et al., ‘‘Infrared photocurrent management in monolithic perovskite/silicon heterojunction tandem solar cells by using a nanocrystalline silicon oxide interlayer,’’ Optic Express, vol. 26, no. 10, p. A487, 2018. Doi.org/10.1364/OE.26.00A487

[S5] K. Jäger, L. Korte, B. Rech, and S. Albrecht, "Numerical optical optimization of monolithic planar perovskite-silicon tandem solar cells with regular and inverted device architectures". *Opt. Express* 25, A473 (2017).

[S6] M. A. Green and M. J. Keevers, ‘‘Optical properties of intrinsic silicon at 300 K,’’ Prog. Photovoltaics Res. Appl., vol. 3, no. 3, pp. 189–192, 1995. doi.org/10.1002/pip.4670030303

[S7] Cruz, A. *et al.* Optoelectrical analysis of TCO$\mathplus$Silicon oxide double layers at the front and rear side of silicon heterojunction solar cells. *Solar Energy Materials and Solar Cells* 236, 111493 (2022).

[S8] Chen, B. et al. Blade-Coated Perovskites on Textured Silicon for 26%-Efficient Monolithic Perovskite/Silicon Tandem Solar Cells. Joule 4, 850–864 (2020).

[S9] Yajie Jiang, Supriya Pillai and Martin A. Green, 'Realistic Silver Optical Constants for Plasmonics', Scientific Reports**, vol. 6, no.** 1, pp.30605, (2016)
